# Effect of Mifepristone vs Placebo for Treatment of Adenomyosis With Pain Symptoms: A Randomized Clinical Trial

**DOI:** 10.1001/jamanetworkopen.2023.17860

**Published:** 2023-06-12

**Authors:** Xuan Che, Jianzhang Wang, Wenting Sun, Jiayi He, Qiming Wang, Danyang Zhu, Weili Zhu, Jing Zhang, Jie Dong, Jingui Xu, Feiyun Zheng, Jianwei Zhou, Weidong Zhao, Qiao Lin, Lingfang Ye, Xiumin Zhao, Zhengfen Xu, Yunyan Chen, Jing Wang, Wenlie Wu, Lingyun Zhai, Yuanyuan Zhou, Jianguang Zheng, Xinmei Zhang

**Affiliations:** 1 Women’s Hospital, School of Medicine, Zhejiang University, Hangzhou, China; 2 Jiaxing University Affiliated Women and Children Hospital, Jiaxing, China; 3 Ningbo Women and Children’s Hospital, Ningbo, China; 4 The First People’s Hospital of Taizhou City, Taizhou, China; 5 The Affiliated Hospital of Medical School of Ningbo University, Ningbo, China; 6 Huzhou Maternity & Child Health Care Hospital, Huzhou, China; 7 Quzhou People’s Hospital, Quzhou, China; 8 The First Affiliated Hospital of Wenzhou Medical University, Wenzhou, China; 9 The Second Affiliated Hospital of Zhejiang University School of Medicine, Hanzhou, China; 10 Anhui Provincial Cancer Hospital, Hefei, China; 11 Taizhou Cancer Hospital, Taizhou, China; 12 Chuangda Pharmaceutical Technology, Shanghai, China

## Abstract

**Importance:**

Adenomyosis is a common chronic gynecological disorder, and its treatment is an unmet need. New therapies need to be developed. Mifepristone is being tested for adenomyosis treatment.

**Objective:**

To determine whether mifepristone is effective and safe for adenomyosis treatment.

**Design, Setting, and Participants:**

This multicenter, placebo-controlled, double-blind randomized clinical trial was conducted in 10 hospitals in China. In total, 134 patients with adenomyosis pain symptoms were enrolled. Trial enrollment began in May 2018 and was completed in April 2019, and analyses were conducted from October 2019 to February 2020.

**Interventions:**

Participants were randomized 1:1 to receive mifepristone 10 mg or placebo orally once a day for 12 weeks.

**Main Outcomes and Measures:**

The primary end point was the change in adenomyosis-associated dysmenorrhea intensity, evaluated by the visual analog scale (VAS) after 12 weeks of treatment. Secondary end points included the change in menstrual blood loss, increased level of hemoglobin in patients with anemia, CA125 level, platelet count, and uterine volume after 12 weeks of treatment. Safety was assessed according to adverse events, vital signs, gynecological examinations, and laboratory evaluations.

**Results:**

In total, 134 patients with adenomyosis and dysmenorrhea were randomly assigned, and 126 patients were included in the efficacy analysis, including 61 patients (mean [SD] age, 40.2 [4.6] years) randomized to receive mifepristone and 65 patients (mean [SD] age, 41.7 [5.0] years) randomized to received the placebo. The characteristics of the included patients at baseline were similar between groups. The mean (SD) change in VAS score was −6.63 (1.92) in the mifepristone group and −0.95 (1.75) in the placebo group (*P* < .001). The total remission rates for dysmenorrhea in the mifepristone group were significantly better than those in the placebo group (effective remission: 56 patients [91.8%] vs 15 patients [23.1%]; complete remission: 54 patients [88.5%] vs 4 patients [6.2%]). All the secondary end points showed significant improvements after mifepristone treatment for menstrual blood loss, hemoglobin (mean [SD] change from baseline: 2.13 [1.38] g/dL vs 0.48 [0.97] g/dL; *P* < .001), CA125 (mean [SD] change from baseline: −62.23 [76.99] U/mL vs 26.89 [118.70] U/mL; *P* < .001), platelet count (mean [SD] change from baseline: −28.87 [54.30]×10^3^/µL vs 2.06 [41.78]×10^3^/µL; *P* < .001), and uterine volume (mean [SD] change from baseline: −29.32 [39.34] cm^3^ vs 18.39 [66.46] cm^3^; *P* < .001). Safety analysis revealed no significant difference between groups, and no serious adverse events were reported.

**Conclusions and Relevance:**

This randomized clinical trial showed that mifepristone could be a new option for treating patients with adenomyosis, based on its efficacy and acceptable tolerability.

**Trial Registration:**

ClinicalTrials.gov Identifier: NCT03520439

## Key Points

**Question:**

Is mifepristone effective for adenomyosis treatment?

**Findings:**

In this randomized clinical trial of 134 participants with adenomyosis pain symptoms, the participants in the mifepristone group and had significantly greater improvement in pain scores than the placebo group. All the secondary outcomes, including hemoglobin, platelet count, and uterine volume, showed significant improvements in the mifepristone group, and there were no serious adverse events.

**Meaning:**

This randomized clinical trial found that mifepristone was effective and safe for the treatment of adenomyosis, which supports the repositioning of this drug as a new treatment option for adenomyosis.

## Teaser

This randomized clinical trial assesses whether mifepristone is effective and safe for the treatment of adenomyosis.

## Introduction

Also known as *internal endometriosis*, adenomyosis is defined as the presence of ectopic endometrial glands and stroma in the myometrium.^1^ The prevalence of adenomyosis has been estimated to range from 5% to 70%, and approximately 20% of reproductive-aged people with a uterus are diagnosed with adenomyosis.^2,3^ The disease causes serious health problems, such as progressive dysmenorrhea, menorrhagia, secondary anemia, enlarged uterus, subfertility, miscarriage, and obstetric complications.^4,5,6^ The health care cost of long-term management for adenomyosis is as high as that of chronic high-impact diseases, such as diabetes and rheumatoid arthritis.^7^ Adenomyosis is an immense clinical challenge in gynecology and health care economics.^8^

At present, medical treatment plays an important role in the management of adenomyosis to relieve the symptoms and retard the progression of the disease.^9^ Unfortunately, there are no approved medical guidelines for adenomyosis. Medical treatment for adenomyosis, including gonadotropin-releasing hormone agonist (GnRH-a), oral contraceptives, and progestins, are limited in terms of effectiveness, tolerability, and costs.^10,11,12^ The current medical treatment strategy for adenomyosis is an unmet medical need.^13,14^ Furthermore, in the US population, 82.0% patients with adenomyosis choose hysterectomy to resolve their adenomyosis-associated symptoms and thereby lose their fertility.^15^ Therefore, there is a need to develop new therapeutic strategies and perform clinical studies focusing on the medical treatment of adenomyosis.

In 1982, mifepristone was reported as the first selective progesterone receptor modulator.^16,17^ Current studies for understanding the mechanism of action of mifepristone are changing our views on the drug beyond its abortifacient scope.^18,19,20^ Mifepristone has been found to have potential clinical applications in gynecological therapies, such as endometriosis and uterine fibroids.^21,22^ A previous study indicated that mifepristone treatment inhibited the development of adenomyosis in mice.^23^ Our previous study^24^ found that mifepristone could downregulate the gene expressions of *CDK1*, *CDK2*, *cyclin B*, *cyclin E*, and *CXCR4* in endometrial epithelial cells to inhibit the proliferation, migration, and invasion of primary endometrial cells in adenomyosis. The study also found that mifepristone treatment decreased the uterine volume and increased hemoglobin for patients with adenomyosis. The expression of the immune checkpoint proteins is downregulated in adenomyosis tissues after mifepristone treatment.^25^ Some small-sample studies in China have reported that mifepristone may be safe and effective for the treatment of adenomyosis. However, it is still controversial whether mifepristone can be used in the treatment of adenomyosis and whether its curative effect is better than that of traditional therapy. To further explore the efficacy of mifepristone for adenomyosis, especially for patients with dysmenorrhea, we conducted a double-blind, multicenter, and placebo-controlled randomized clinical trial.

## Methods

### Trial Design and Oversight

This randomized clinical trial was initiated and designed by the investigators and performed in accordance with the principles of the Declaration of Helsinki and the guidelines of Good Clinical Practice. The study protocol and statistical analysis plan are provided in Supplement 1. Ethical approval was obtained by the institutional review board or ethics committee at each investigational site before trial initiation. Each patient provided written informed consent before the initiation of trial-specific procedures. Data analysis and reporting were performed in accordance with the Consolidated Standards of Reporting Trials (CONSORT) reporting guideline.

The trial was conducted in 10 hospitals in China (eAppendix 1 in Supplement 2). Trial enrollment began on May 19, 2018, and was completed on April 4, 2019. Patients were enrolled by their clinicians. The investigators conducted the trials, gathered the data, inputted the data, and maintained the trial database using Electronic Data Capture (EDC) System in a blinded manner. Zizhu Pharmaceutical provided trial drugs but did not participate in any part of the study. The drugs were prelabeled on the package by the investigators who did not participate in any part of the trial. Chuangda Pharmaceutical Technology provided the data management and statistical analysis.

### Trial Participants

Eligible patients included premenopausal female patients aged between 18 and 50 years with a diagnosis of adenomyosis confirmed by designated ultrasonography or magnetic resonance imaging (MRI) specialists. Patients with adenomyosis-associated dysmenorrhea (visual analog scale [VAS] score >0 points) were included with or without menorrhagia (Pictorial Blood Loss Assessment Chart [PBAC] score ≥100 points). In consideration of the safety of patients in the trial, patients with severe anemia were excluded. The key exclusion criteria were a history of undiagnosed vaginal bleeding outside reference ranges, endometrial malignant tumors, uterine fibroids, or endometriosis. To maintain masking and avoid unwanted pregnancy, patients who did not agree to use nonhormonal contraceptive methods (ie, contraceptive barriers, such as condoms) were excluded. The details are provided in the study protocol (Supplement 1 and eAppendix 2 in Supplement 2).

### Trial Procedures

After 4 weeks of screening, patients with adenomyosis were randomly assigned at a 1:1 ratio to receive mifepristone (10 mg) or placebo orally once a day for 12 weeks during the double-blind period. Regular trial visits were performed every 4 weeks and lasted until 4 weeks after treatment. The efficacy of treatment, adverse events, and safety assessments were all recorded throughout the trial. Patients were not allowed to use other drugs or treatment methods that affect the efficacy evaluation. From an ethical point of view, it is considered acceptable for patients to receive analgesics in placebo-controlled trials, and the use of indomethacin suppositories is recommended only to avoid trial bias.

### Randomization and Blinding

Eligible patients were randomly assigned at a 1:1 ratio to receive mifepristone or placebo. The randomization sequence was generated by a statistician using the block-rank package in R software version 3.4.1 (R Project for Statistical Computing) and was stratified by center with a block size of 4. Randomization was performed centrally with an interactive web response system. The participant and drug numbers were all unique and corresponded to one another and were handled independently by the other designated statistician. Additionally, confidentiality was maintained for the clinician and patients. None of the statisticians participated in any part of the trial.

The placebo tablets had the same appearance (size, shape, and color) as the mifepristone tablets. The route and administration of the assigned intervention were identical in the 2 groups. When a patient submitted written informed consent and agreed to participate in the trial, the interactive web response system temporarily generated a corresponding drug code, and then the drug with the code was allocated to the patient. Allocation concealment was maintained by blinding the patients, investigators, clinicians, and statisticians until the study was completed.

### End Points

The primary efficacy end point was the change in adenomyosis-associated dysmenorrhea intensity, evaluated using the VAS, from baseline to 12 weeks of treatment. The VAS score ranged from 0 to 10, and a higher score represented greater pain intensity. The complete remission and total effective rates for dysmenorrhea were evaluated. The complete remission rate was defined as the proportion of patients who achieved a complete remission after treatment. The total efficacy rate was defined as the proportion of patients with VAS scores reduced by at least 30% from baseline to treatment completion.

The first key secondary end point was the change in menstrual blood loss volume from baseline to 12 weeks in patients with heavy menstrual bleeding, including the complete remission rate and total efficacy rate for menstrual blood loss. Menstrual blood loss was analyzed with the PBAC, which is considered a valid tool with high specificity and sensitivity.^26^ A PBAC score of 100 means more than 80 mL of blood loss, which was defined as heavy menstrual bleeding. The complete remission rate for heavy menstrual bleeding was defined as the proportion of patients who achieved amenorrhea and the total efficacy rate for menstrual blood loss was defined as the proportion of patients with PBAC scores reduced by at least 30% from baseline to treatment completion. The second key secondary end point was the mean change in hemoglobin levels. Additional secondary end points were the changes in CA125 levels, platelet counts, and uterine volume. Uterine volume was calculated with the following formula: 0.52 × *length* × *anteroposterior diameter* × *transverse diameter*.^27^

Safety evaluations, including the incidence and severity of adverse events, were collected throughout the trial. Safety evaluations included various concerns after drug treatment, vital signs, endometrial biopsies, laboratory evaluations, and physical and gynecological examinations (especially changes in endometrial thickness, liver parameters, and follicle-stimulating hormone [FSH] and luteinizing hormone [LH] levels).

### Statistical Analysis

We calculated that a sample of 60 participants per group would provide at least 90% power with a .05 2-sided significance. According to the conservative principle in the sample size calculation, the decrease in the VAS score was assumed to be 5.24 in the placebo group and 6.48 in the mifepristone group (eAppendix 3 in Supplement 2).

Efficacy was analyzed in the full-analysis set (FAS) and per-protocol set (PPS). The FAS population included eligible participants who were randomized, and patients without any valid data at baseline were excluded. The PPS population was defined as all randomized patients who received at least 1 dose of the investigational product and had valid VAS assessments at and after baseline. The primary analysis was performed. Missing values were excluded from the analysis without missing value imputation. Furthermore, a sensitivity analysis of the primary efficacy outcome was performed to assess the effect of discontinuation under different missing data mechanisms. Missing values for continuous outcomes were handled using a multiple imputation method with fully conditional specification. Trial group, age, and the results of other visit time points, such as VAS score, were used as covariates, and 50 imputed data sets were generated. After multiple imputation was been performed, the next steps were to apply statistical tests in each imputed data set and to pool the results to obtain summary estimates. Adverse events were analyzed in the safety set, defined as participants who received at least 1 dose of the intervention or placebo drug.

As the primary end point, the change in VAS score from baseline to week 12 between groups was compared using the Wilcoxon test. The Pearson χ^2^ test was performed for the comparison of the total efficacy rate for dysmenorrhea and complete remission of dysmenorrhea. The secondary end points were compared using an independent sample *t* test, Pearson χ^2^ test, or Fisher exact test, as appropriate. When continuous variables did not follow a Gaussian distribution, they were presented as the median (IQR), and the groups were compared using Mann-Whitney *U* test. All statistical analyses were conducted using SAS software version 9.3 (SAS Institute). Statistical significance was defined as *P* < .05 with 2-sided testing. Data were analyzed from October 2019 to April 2022.

## Results

### Participants

A total of 194 patients were screened, of whom 134 were eligible for participation and underwent randomization at a 1:1 ratio, with 66 patients assigned to the mifepristone group and 68 patients assigned to the placebo group (eFigure 1 in Supplement 2). Eight patients (5.9%) were lost to follow-up (5 patients in the mifepristone group and 3 patients in the placebo group). The FAS population was defined as all randomized patients with valid data at baseline, including 61 patients (mean [SD] age, 40.2 [4.6] years) in the mifepristone group and 65 patients (mean [SD] age, 41.7 [5.0] years) in the placebo group. All patients in the FAS population were included in efficacy analysis. The characteristics of included patients at baseline were similar between groups (Table 1).

**Table 1.** Baseline Characteristics of Study Participants

Characteristics	Mean (SD)	
	Mifepristone (n = 61)	Placebo (n = 65)
Age, y	40.2 (4.6)	41.7 (5.0)
BMI	23.0 (2.3)	22.7 (2.9)
Gravidity, No.	3.0 (1.4)	3.0 (1.6)
Parity, No.	1.4 (0.6)	1.2 (0.6)
Abortion, No.	1.6 (1.3)	1.8 (1.4)
VAS score^a^	6.7 (1.8)	6.2 (2.1)
Menstrual blood loss, median (IQR)^b^	135 (72-216)	114 (59-212)
Menses duration, d	7.1 (2.6)	6.7 (2.3)
Menstrual cycle, d	30.9 (6.3)	30.5 (6.0)
Patients with heavy menstrual bleeding, No. (%)	40 (65.6)	37 (56.9)
Uterine volume, cm^3^^c^	156.5 (58.9)	170.2 (89.6)
Hemoglobin level, g/dL	11.32 (1.54)	11.54 (1.62)
Patients with anemia, No. (%)^d^	36 (59.0)	35 (53.8)
Platelet count, ×10^3^/μL	273.2 (84.0)	269.7 (58.0)
CA125, U/mL, median (IQR)	68.6 (34.4-111.2)	67.9 (36.9-104.3)
Endometrial thickness (monolayer), cm^e^	0.4 (0.1)	0.3 (0.1)

Abbreviations: BMI (calculated as weight in kilograms divided by height in meters squared); VAS, visual analogue scale.

SI conversion factors: To convert hemoglobin to grams per liter, multiply by 10; platelet count to ×10^9^/L, multiply by 1.

^a^ VAS was used to evaluate the intensity of adenomyosis-associated dysmenorrhea. Score ranged from 0 to 10, and a higher score represented a greater intensity of pain.

^b^ The menstrual blood loss was analyzed with the Pictorial Blood Loss Assessment Chart, with a score of 100 indicating with more than 80 mL of blood loss, which was defined as heavy menstrual bleeding.

^c^ Uterine volume was determined based on the diameters of 3 angles by transvaginal ultrasonography. The uterine volume was calculated as 0.52 × *length* × *anteroposterior diameter* × *transverse diameter*.

^d^ Anemia at baseline was defined as having a less than 12.0 g/dL hemoglobin.

^e^ Endometrial thickness was detected by transvaginal ultrasonography.

### Primary Efficacy End Point

The results of the efficacy outcomes in the FAS population are shown in Table 2. The mean (SD) VAS scores at baseline were 6.7 (1.8) points in the mifepristone group and 6.2 (2.1) points in placebo group (*P* = .21) (Table 1). After 12 weeks of treatment, the mean (SD) changes in VAS scores from baseline were −6.63 (1.92) points in the mifepristone group and −0.95 (1.75) points in the placebo group (between-group difference, −5.68; 95% CI, −6.37 to −4.99; *P* < .001) (Table 2).

**Table 2.** Summary of Primary and Secondary End Points at Week 12 in the Full Analysis Population

End point	Mean (SD)		Difference (95% CI)^a^	*P* value
	Mifepristone (n = 61)	Placebo (n = 65)		
Primary: change in VAS score from baseline^b^	−6.63 (1.92)	−0.95 (1.75)	−5.68 (−6.37 to −4.99)	<.001
Secondary				
The complete remission rate for heavy menstrual bleeding, No. (%)^c^^,^^d^	36 (90.00)	2 (5.41)	84.60 (72.80 to 96.40)	<.001
Total effective rate for heavy menstrual bleeding, No. (%)^e^	38 (95.00)	14 (37.84)	57.2 (40.10 to 74.20)	<.001
The complete remission rate for anemia, No. (%)^f^	28 (77.78)	8 (22.85)	53.5 (33.6 to 73.5)	<.001
Change from baseline in Hb, g/L	2.13 (1.38)	0.48 (0.97)	1.64 (1.05 to 2.24)	<.001
Change from baseline in CA125, U/mL	−62.23 (76.99)	26.89 (118.70)	−89.12 (−133.12 to −45.11)	<.001
Change from baseline in platelet count, 10^3^/μL	−28.87 (54.30)	2.06 (41.78)	−30.93 (−49.93 to −11.92)	<.001
Change from baseline in uterine volume, mean (SD), cm^3^^g^	−29.32 (39.34)	18.39 (66.46)	−47.71 (−69.18 to −26.25)	<.001

Abbreviations: Hb, hemoglobin; VAS, visual analogue scale.

SI conversion factors: To convert Hb to grams per liter, multiply by 10; platelet count to ×10^9^/L, multiply by 1.

^a^ Differences for continuous end points are shown as means, and differences for categorical end points are shown in percentage points.

^b^ VAS scores range from 0 to 10, with higher scores indicating more severe dysmenorrhea.

^c^ Definition of heavy menstrual bleeding is more than 100 of the Pictorial Blood Loss Assessment Chart score.

^d^ Definition of the complete remission rate for heavy menstrual bleeding is the percentage of patients achieving amenorrhea after 12 weeks treatment.

^e^ Definition of total effective rate for heavy menstrual bleeding is the percentage of patients with reduction in Pictorial Blood Loss Assessment Chart score of at least 30% from baseline.

^f^ Definition of anemia patients at baseline is the condition of having hemoglobin levels less than 12.0 g/dL quantity.

^g^ Uterine volume was determined based on the diameters of 3 angles by transvaginal ultrasonography. The uterine volume was calculated as 0.52 × *length* × *anteroposterior diameter* × *transverse diameter*.

**Figure 1.**
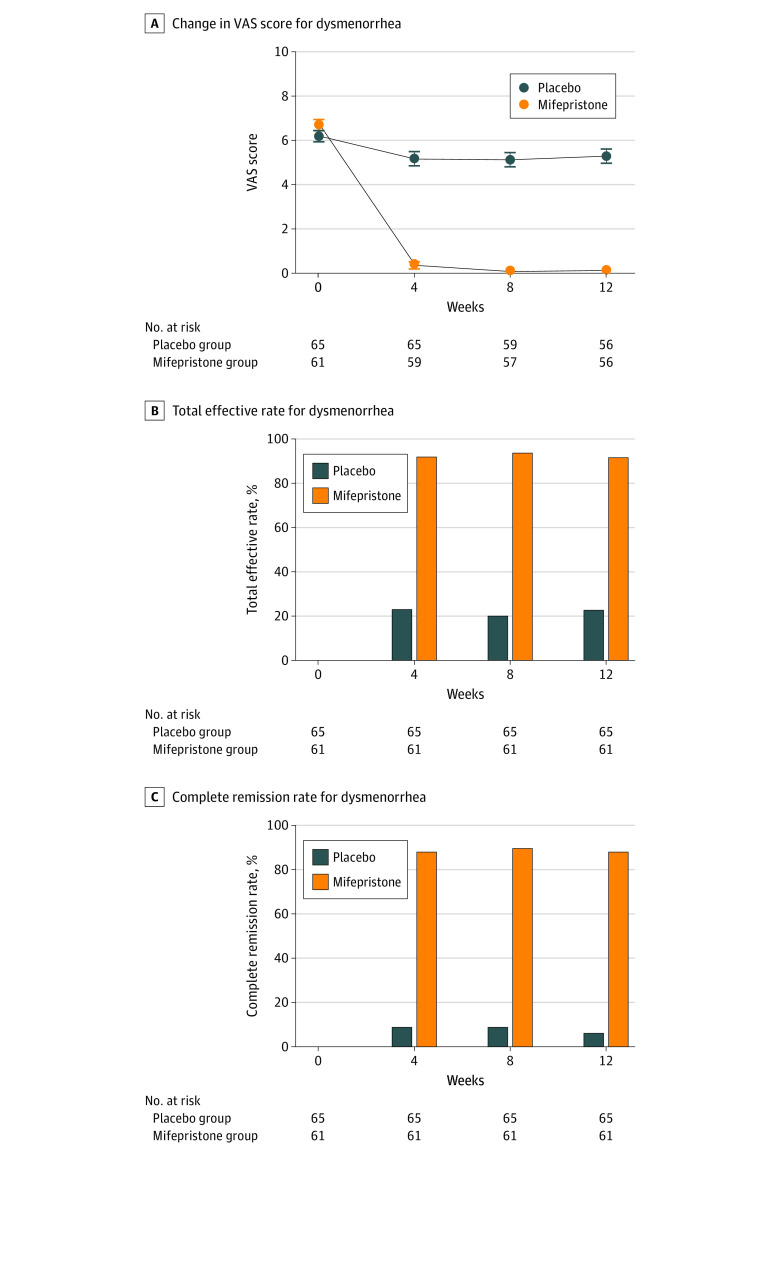
The Effect of Mifepristone on Dysmenorrhea in Patients With Adenomyosis Adenomyosis-associated dysmenorrhea intensity was evaluated by the visual analogue scale (VAS) score (range, 0-10; a higher score indicates worse symptom severity).

In fact, the VAS score significantly began to decrease at week 4 after mifepristone treatment (Figure 1A). Furthermore, both the total efficacy and complete remission rates for dysmenorrhea in the mifepristone group were significantly greater than those in placebo group at week 12 (total efficacy: 56 patients [91.8%] vs 15 patients [23.1%]; *P* < .001; complete remission: 54 patients [88.5%] vs 4 patients [6.2%]; *P* < .001) (Figure 1B and C). The results of the sensitivity analysis for missing data using the multiple imputation method were similar to those of the primary analysis of the FAS population (eTables 1-3 in Supplement 2).

### Key Secondary End Points

**Figure 2.**
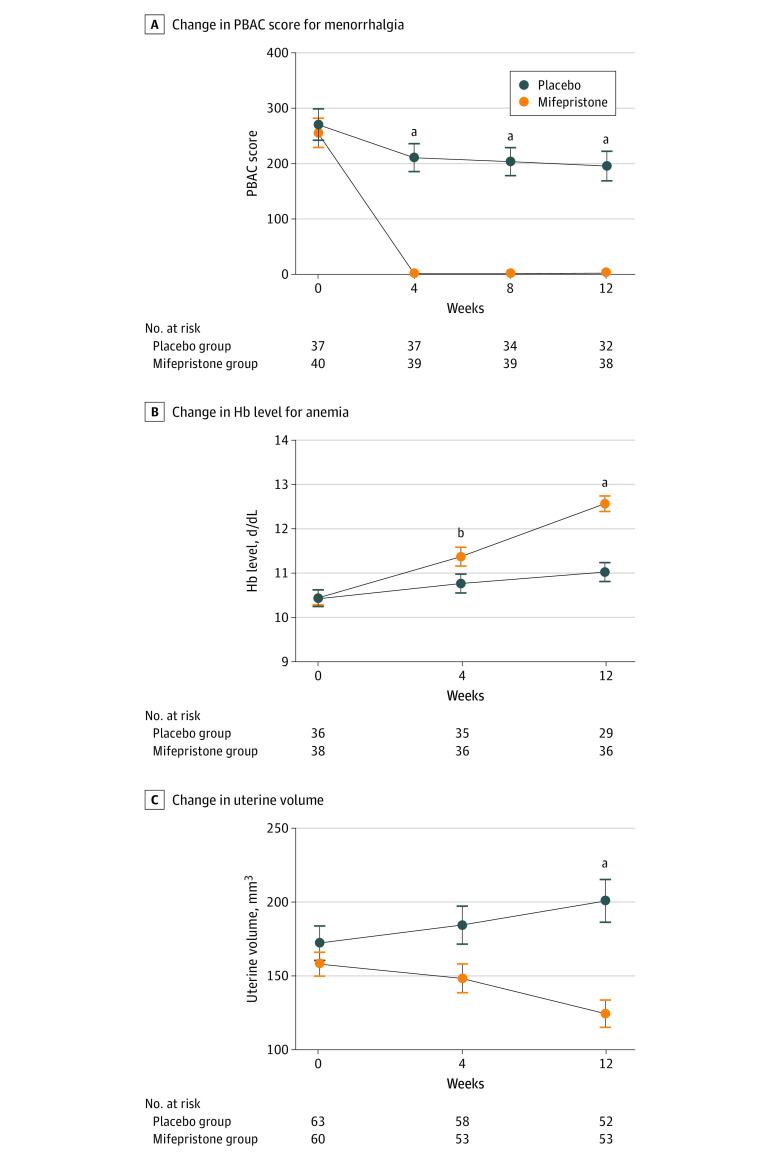
Key Secondary Outcomes After Mifepristone Treatment Hb indicates hemoglobin; PBAC, Pictorial Blood Loss Assessment Chart. To convert Hb to grams per liter, multiply by 10.

All the secondary outcomes showed significant improvements in mifepristone group compared with placebo group (Table 2). There was a significant reduction in the change in PBAC for menorrhagia after mifepristone treatment (Table 2 and Figure 2A; eTable 4 in Supplement 2). The mifepristone group, compared with the placebo group, had significantly improved complete remission for heavy menstrual bleeding (36 patients [90.00%] vs 2 patients [5.41%]) and total efficacy for heavy menstrual bleeding (38 patients [95.00%] vs 14 patients [37.84%]) after 12 weeks of treatment. The results of the sensitivity analysis for menstrual bleeding in FAS population and for efficacy outcomes in PPS population were all similar to the results in the FAS population (eTable 5 and eTable 6 in Supplement 2).

Mifepristone therapy resulted in significant improvements in hemoglobin levels in patients with anemia (Figure 2B). The complete remission rates for anemia were 28 patients (77.78%) in the mifepristone group and 8 patients (22.85%) in the placebo group (*P* < .001) (Table 2). Among patients with anemia, hemoglobin levels increased by a mean (SD) of 2.13 (1.38) g/dL (to convert to grams per liter, multiply by 10) in the mifepristone group and 0.48 (0.97) g/dL in the placebo group at week 12 (*P* < .001) (Table 2).

Other key efficacy end points also showed significant improvements in the mifepristone group (Table 2). The mean (SD) change in CA125 levels from baseline to week 12 was −62.23 (76.99) U/mL in the mifepristone group and 26.89 (118.70) U/mL in the placebo group (*P* < .001). The mean (SD) change in platelet count from baseline was −28.87 (54.30)×10^3^/µL (to convert to ×10^9^/L, multiply by 1) in the mifepristone group and 2.06 (41.78) ×10^3^/µL in the placebo group (*P* < .001). In particular, uterine volume was significantly decreased after 12 weeks of mifepristone treatment compared with placebo (Figure 2C). The mean (SD) change in uterine volume from baseline was −29.32 (39.34) cm^3^ in the mifepristone group and 18.39 (66.46) cm^3^ in the placebo group (*P* < .001) (Table 2). The results of the primary analysis for these key efficacy outcomes in PPS population were similar to the results in the FAS population (eTable 7 in Supplement 2).

### Safety and Adverse Events

Adverse events were recorded in 18 patients (30.51%) in the mifepristone group and 14 patients (21.88%) in the placebo group, and no statistical difference was observed (Table 3). Adverse events are listed in Table 3. No serious adverse events were reported. All adverse events were considered mild or moderate, such as mildly elevated levels of liver aminotransferases (eTable 8 and eTable 9 in Supplement 2). None of the patients in mifepristone group discontinued in the trial, and no patient in either group had dose reduction.

**Table 3.** Adverse Events in the Safety Population^a^

Adverse Event	Patients, No. (%)	
	Mifepristone (n = 59)	Placebo (n = 64)
Any adverse event	18 (30.51)	14 (21.88)
Serious adverse events^b^	0	0
Any adverse event leading to trial-drug discontinuation	0	4 (6.26)
Adverse events		
Back pain	1 (1.69)	0
Constipation	0	1 (1.56)
Hot flashes	5 (8.47)	1 (1.56)
Nausea	2 (3.39)	3 (4.69)
Fatigue	1 (1.69)	1 (1.56)
Pneumonia	0	1 (1.56)
Somnolence	1 (1.69)	1 (1.56)
Vomiting	0	1 (1.56)
Breast tenderness	1 (1.69)	1 (1.56)
Weight gain	4 (6.78)	1 (1.56)
Headache	1 (1.69)	0
Stomach discomfort	1 (1.69)	1 (1.56)
Throat pain	0	1 (1.56)
Dazzling	1 (1.69)	0
Metrorrhagia	2 (3.39)	1 (1.56)
Increased vaginal discharge	0	1 (1.56)

^a^ Safety population was defined as the participants with at least 1 dose of the intervention or placebo drug. Adverse events were included with an onset date on or after the date of first dose and up to and including 28 days following the date of last dose of study medication.

^b^ A serious adverse event was defined as any adverse event that resulted in death, was immediately life-threatening, led to inpatient hospitalization or prolongation of hospitalization, caused persistent or clinically significant disability or incapacity, or resulted in a congenital anomaly or birth defect.

The results of FSH, LH, and E2 levels analysis showed no significant differences between groups after 12 weeks of treatment (eFigure 2 in Supplement 2). The endometrial thickness detected on ultrasonography after mifepristone treatment had no tendency to increase and, instead, even tended to be thin (eFigure 3 and eTables 10-12 in Supplement 2). No endometrial hyperplasias or cancers were found in the trial. There were no significant differences in body mass index between groups after 12 weeks of treatment (eFigure 3 in Supplement 2).

## Discussion

To our knowledge, this study is the first double-blind randomized clinical trial of mifepristone for adenomyosis treatment. This study included a placebo-controlled and multicenter design, strict randomization and masking with high adherence to treatment, and similar baseline characteristics between groups, thus providing strong evidence.

The result showed that adenomyosis-associated dysmenorrhea intensity was significantly reduced after mifepristone treatment. Regardless of how severe the intensity of dysmenorrhea, the total efficacy for dysmenorrhea was satisfactory. The secondary end point assessments were all significantly improved after 12 weeks of treatment. Most patients with anemia completely recovered to levels within reference range after 12 weeks of treatment.

Drug research and development in adenomyosis has been slow. GnRH-a is markedly efficient in controlling dysmenorrhea and menorrhagia and reducing uterine size for adenomyosis.^28,29^ However, the acceptability of and adherence to GnRH-a treatment are much lower than expected owing to its high cost and high rates of adverse effects and bone resorption.^30^ In this study, mifepristone was as effective, if not more effective than GnRH-a, in controlling dysmenorrhea.^31^ Furthermore, mifepristone has a relatively low price for long-term treatment. In the future, we plan to perform a parallel-controlled randomized clinical trial to further investigate the comparison between mifepristone and GnRH-a for the treatment of adenomyosis.

As an older drug, previous evidence show that mifepristone treatment is well tolerated in long-term administration.^28,29^ In this trial, no serious adverse events were observed, except mild liver function anomalies. GnRH-a downregulates the pituitary-ovarian-gonadal axis to treat adenomyosis.^30,31^ Estrogen deficiency restricts the long-term use of GnRH analogs for premenopausal symptoms.^32^ Currently, the effects of mifepristone on sex hormones remain unclear.^33^ We found no statistically significant differences in FSH, LH, and E2 levels between groups for the 12-week treatment. Recently, the effects of selective progesterone receptor modulators on endometrial thickness and endometrial changes associated with progesterone receptor modulators have attracted much attention but remain controversial.^34,35,36^ Our study showed that endometrial thickness detected by ultrasonography after mifepristone treatment was thinner.

### Limitations

This study has some limitations. First, this trial was conducted only in China. Second, endometrial biopsy was not performed, owing to ethical principles, and no obvious anomalies in the endometrial thickness of patients were detected by ultrasonography. Third, this trial was limited to 12 weeks of treatment, and future research to assess the long-term effects of mifepristone therapy for adenomyosis is still needed.

## Conclusions

In this placebo-controlled randomized clinical trial of mifepristone vs placebo for the treatment of adenomyosis, 12 weeks of mifepristone treatment at a dose of 10 mg per day led to a significant remission for patient symptoms with acceptable tolerability. Therefore, mifepristone was effective and safe for the treatment of adenomyosis. However, further research is still needed to determine the long-term safety and efficacy.

## Notes

Supplement 1.
Trial Protocol and Statistical Analysis Plan

Supplement 2. **eAppendix 1.** Study Sites and Principal Investigators **eAppendix 2.** Inclusion Criteria and Exclusion Criteria **eAppendix 3.** Method of Sample Size Calculation **eTable 1.** Change in VAS After Mifepristone Treatment **eTable 2.** Total Effective Rate for Dysmenorrhea After Mifepristone Treatment **eTable 3.** Complete Remission Rate for Dysmenorrhea After Mifepristone Treatment **eTable 4.** Change in PBAC for Heavy Menstrual Bleeding After Mifepristone Treatment **eTable 5.** Total Effective Rate for Heavy Menstrual Bleeding After Mifepristone Treatment **eTable 6.** Complete Remission Rate for Heavy Menstrual Bleeding After Treatment **eTable 7.** Changes From Baseline in Hb, CA125, PLT and Uterine Volume After Mifepristone Treatment **eTable 8.** Relevant Laboratory Evaluations Changed From Normal to Abnormal After Mifepristone Treatment **eTable 9.** Changes in Liver Function in the 2 Groups During Treatment **eTable 10.** Comparison of the Changes of Endometrial Thickness Between the 2 Groups After 4 Weeks of Treatment **eTable 11.** Comparison of the Change of Endometrial Thickness Between the 2 Groups After 12 Weeks of Treatment **eTable 12.** Comparison of the Endometrial Thickness Between the 2 Groups During the Treatment **eFigure 1.** Flowchart of Study Enrollment **eFigure 2.** Levels of FSH, LH and E2 Between the 2 Groups at Different Periods of Treatment **eFigure 3.** Changes in Endometrial Thickness and BMI Between the 2 Groups at Different Periods of Treatment

Supplement 3.
Data Sharing Statement
