# Supplementary material for: Effect of Mifepristone vs Placebo for Treatment of Adenomyosis With Pain Symptoms: A Randomized Clinical Trial
Source: JAMA Netw Open. 2023 Jun 12;6(6):e2317860. doi: 10.1001/jamanetworkopen.2023.17860 (PMC10261993; doi:10.1001/jamanetworkopen.2023.17860)
Supplement: Supplement 1. — Trial Protocol and Statistical Analysis Plan [file jamanetwopen-e2317860-s001.pdf]

Protocol ID: MF2018
Version：1.0
Date：2018.1.20

Multi-center, Randomized, Double-blind, Controlled Study of the Effectiveness and Safety of Mifepristone in Treating Adenomyosis

Clinical Research Department: Women's Hospital, School of Medicine Zhejiang University

Project Leader: Professor Zhang Xinmei

Discussion time: January 20, 2018

The Department of Data Management and Statistics: Chuangda Pharmaceutical Technology (Shanghai) Co., Ltd.

Cooperative Research Organization: Chuangda Pharmaceutical Technology (Shanghai) Co., Ltd.

Trial drug supplier: China Resources Zizhu Pharmaceutical Co., Ltd.

Sponsor: Women's Hospital, School of Medicine Zhejiang University

---

Privacy statement: this trial plan is classified as confidential information, which is used to be provided for medical experts related to the trial, the relevant staff such as researchers participating in the trial, and medical institutions, ethical committees, and cooperative research organizations and other relevant businesses commissions that undertake the trial. Except for the circumstances described to the subject, no part of the protocol may be disclosed or divulged to a third party without the prior written consent of the sponsor. In addition, when some or all of the results of this clinical trial are published externally to institutions, magazines, etc., they need the written consent of the sponsor.

Multi-center, Randomized, Double-blind, Controlled Study of the Effectiveness and Safety of Mifepristone in Treating Adenomyosis

Names of hospitals participating in clinical trials, principal investigators of each center

Names of hospitals participating in clinical trials (No. of the center)	Principal investigators of each center
Women's Hospital, School of Medicine Zhejiang University (01)	Xinmei Zhang
Anhui Province Cancer Hospital (02)	Weidong Zhao
Huzhou Maternity and Child Care Hospital (03)	Dong Jie
Jiaxing Maternity and Child Health Care Hospital (04)	Weili Zhu
Jinhua Municipal Central Hospital (05)	Hu Min
The Central Hospital of Lishui City (06)	Hongyou Zhou
The Affiliated Hospital of Medical School of Ningbo University (07)	Zhang Jing
Ningbo Maternity and Child Care Hospital (08)	Qiming Wang
The People's Hospital of Quzhou City (09)	Jingui Xu
Shaoxing Women and Children's Hospital (10)	Yungen Wang
Shaoxing People's Hospital (11)	Haigang Ding
The First Affiliated Hospital of Wenzhou Medical University (12)	Feiyun Zheng
The Second Affiliated Hospital of Zhejiang University School of Medicine (13)	Jianwei Zhou
Sir Run Run Shaw Hospital, Zhejiang University School of Medicine (14)	Guanghui Song
Taizhou First People's Hospital (15)	Zhu Danyang
Zhoushan Maternity and Infant Hospital (16)	Shi Xiao

Signature of the medical officer of the sponsor:

The signature indicates agreement with the contents of the final version of the clinical research protocol.

Name:
Responsibilities:

Date: Signature:

Researcher's signature:

The signature indicates agreement with the contents of the final version of the clinical research protocol.

Name: Zhao Weidong
Department: Anhui Province Cancer Hospital Signature:

Name: Dong Jie
Department: Huzhou Maternity and Child Care Hospital Signature:

Name: Zhu Weili
Department: Jiaxing Maternity and Child Health Care Hospital Signature:

Name: Hu Min
Department: Jinhua Municipal Central Hospital Signature:

Name: Zhou Hongyou
Department: The Central Hospital of Lishui City Signature:

Name: Zhang Jing
Department: The Affiliated Hospital of Medical School of Ningbo University
Signature:

Name: Wang Qiming
Department: Ningbo Maternity and Child Care Hospital Signature:

Name: Xu Jingui
Department: The People's Hospital of Quzhou City Signature:

Name: Ding Haigang
Department: Shaoxing Women and Children’s Hospital Signature:

Name: Wang Yungen
Department: Shaoxing People’s Hospital Signature:

Name: Zheng Feiyun
Department: The First Affiliated Hospital of Wenzhou Medical University

Signature:

Multi-center, Randomized, Double-blind, Controlled Study of the Effectiveness and Safety of Mifepristone in Treating Adenomyosis

Name: Zhou Jianwei
Department: The Second Affiliated Hospital of Zhejiang University School of Medicine Signature:

Name: Zhang Xinmei
Department: Women’s Hospital, School of Medicine Zhejiang University Signature:

Name: Song Guanghui
Department: Sir Run Run Shaw Hospital, Zhejiang University School of Medicine Signature:

Name: Zhu Danyang
Department: Taizhou First People’s Hospital Signature:

Name: Shi Xiao
Department:：Zhoushan Maternity and Infant Hospital Signature:

The program abstract

**Research topic**	**Multi-center, Randomized, Double-blind, Controlled Study** of the Effectiveness and Safety of Mifepristone in Treating Adenomyosis
**Trial objective**	To investigate the effectiveness and safety of mifepristone in treating adenomyosis
**Trial drug**<br>**Active ingredient**<br>**Dosage**<br>**Approach**<br>**Time**	Mifepristone tablets<br>Mifepristone<br>10 mg daily<br>Orally<br>Twelve weeks
**Trial drug**<br>**Active ingredient**<br>**Dosage**<br>**Approach**<br>**Time**	Placebo<br>N/A<br>N/A<br>One tablet daily, orally<br>The longest 12 weeks
**Indication**	Adenomyosis meeting the criteria
**Research design**	Multi-center, randomized, double-blind, allocation concealment, placebo-controlled, parallel grouping
**The type of control**	12 weeks of placebo control
**Inclusion criteria**	1. Women had a diagnosis of adenomyosis by B-ultrasound or pelvic MRI examination and the volume of their uterus were less than those with 10 weeks of pregnancy<br>2. Women with 10-cm visual analogue scale (VAS) of adenomyosis-associated pain > 0 point with or without menorrhagia (PBAC≥100 points)<br>3. Women between 18 and 50 years of age who currently have no childbearing requirement

	4. Women who had normal cervical cytology test<br>5. Women who agreed to choose a barrier method of contraception<br>6. Women who agreed to adhere to the study requirements and signed the informed consent<br>7. Women who were not postmenopausal
**Exclusion criteria**	1. Patients with HB < 90G/L<br>2. Patients with undiagnosed abnormal vaginal bleeding or endometrial lesions<br>3. Patients who were preparing to give birth when taking the medication or within 6 months of stopping the medication<br>4. Patients with malignant tumors (including the reproductive system and other systems);<br>5. Patients with severe heart, liver, kidney disease and adrenocortical insufficiency<br>6. Patients who had the results of follow-up laboratory test indicating abnormal clinical significance<br>7. Patients who had a known allergy to mifepristone, or related compounds<br>8. Patients with any other diseases or conditions that may cause the study drug to alter absorption, accumulate excessively, affect metabolism, or change the excretion pattern<br>9. Patients with clinically significant depression within the current or most recent year<br>10. Patients who regularly took analgesics due to other underlying diseases<br>11. Patients used the drugs, such as Ketoconazole, itraconazole, erythromycin, rifampicin, corticosteroids (hydrocortisone, prednisone, dexamethasone, etc.), and some anticonvulsants

	(phenytoin, phenobarsbital, carbamazepine, etc.), griseofulvin, non-steroidal anti-inflammatory drugs (aspirin, acetaminophen, etc.) and couldn't be stopped during the study<br>12. Patients currently used any other hormone drugs, including: a. use of GnRH agonists within 6 months before the screening period; b. use of progestins or danazol and other long-acting hormones within 3 months before the screening period; c. use of oral contraceptive-like short-acting hormones within one month before the screening period;<br>13. Patients who participated in other clinical trials within 3 months before the screening, or who were considered inappropriate to participate in the study by other investigators.
**Number of subjects**	Subjects are screened and 150 subjects are randomized into groups.
**Safety indicator**	(1) General observation items: gynecological examination, blood pressure, heart rate, weight<br>(2) Auxiliary examination: pelvic ultrasonography (uterine volume, endometrium), magnetic resonance (non-essential), breast B ultrasound, sex hormones, blood routine, coagulation function, liver and kidney function, blood lipid, blood/urine pregnancy test, and endometrial biopsy (if necessary)<br>(3) Adverse Event Monitoring: adverse events, drug side effects and adverse drug reactions
**Effectiveness indicator**	(1) Changes in chronic pelvic pain associated with adenomyosis (VAS scale)<br>(2) Changes in uterine bleeding (PBAC scale)<br>(3) Improvement of anemia (blood routine)<br>(4) Size of the uterus

Abbreviations

Abbreviation	Name
ALT	Alanine aminotransferase
APTT	Activated partial thromboplastin time
AST	Aspartate aminotransferase
BUN	Blood urea nitrogen
Cr	Creatinine
E2	Estradiol
FSH	Follicular estrogen hormone
HB	Hemoglobin
HCG	Human chorionic gonadotropin
HDL	High-density lipoprotein
LH	Luteinizing hormone
N/A	Not Applicable
PBAC	Pictorial blood loss assessment chart
PLT	Platelet count
PT	Prothrombin time
RBC	Red blood cell count
TBIL	Total bilirubin
TC	Total cholesterol
TG	Triglyceride
VAS	Visual analogue scale

Multi-center, Randomized, Double-blind, Controlled Study of the Effectiveness and Safety of Mifepristone in Treating Adenomyosis

Catalogue

Names of hospitals participating in clinical trials and principal investigators of each center I

Signature of the medical officer of the sponsor: II

Researcher's signature: III

The program abstract V

1. Introduction 1

1.1 Research background 1
1.2 Research principle 1
1.3 Benefit - risk analysis 1

2. Research purposes 2

3. Researchers and other research participants 2

4. Research design 3

4.1 Overview of research design 3
4.2 The sketch map of research design 3
4.3 Rationality of research design 3
4.4 End of study 4

5. Study population 4

5.1 Inclusion criteria 4
5.2 Exclusion criteria 4
5.3 Quit research 5
5.4 Subject replacement 6
5.5 Subject number 7

6. Research drug and administration 6

6.1 Drugs to be given 6
6.2 Treatment grouping 6
6.3 The package of trial drug 7
6.4 Distribution, preservation and recycle of research drug 7
6.5 Administration of the research drug 7
6.6 Blind method 9
6.7 Post-study treatment 9
6.8 Combining medication and treatment 10

7. Research steps and variables 9

7.1 Research steps 9
7.2 Effectiveness 15
8. Safety indicators 15
9. Quality control and guarantee 16
10. The methods of observation, record and report of adverse events 16
10.1 Adverse events 16
10.2 Serious adverse events 17
10.3 Medical side effects 17
10.4 Severity of adverse reactions 17
10.5 Judgment of causality between adverse reactions and drugs 17
10.6 Adverse event handling 18
11. Study medical records, ecrf filling and transfer 19
12. The relevant requirements of observation, record and summary 19
13. Medical ethics requirements and protection of subjects' rights and interests 19
14. Subject's medication compliance 19
15. Data management and statistical analysis 19
15.1 Monitoring of data records 19
15.2 Data entry and modification 21
15.3 Data locking 22
15.4 Selection of statistical analysis data 22
15.5 Statistical analysis method 22
15.6 The monitoring system 24
16. The management of clinical trial drug 24
16.1 Trial drug packaging 24
16.2 Trial drug distribution 25
16.3 Trial drug inventory 25
16.4 Trial drug preservation 25
17. Modification of the program 26
18. Summary and preservation of data 26
18.1 Data summary 26
18.2 Data preservation 26
19. References 26

1. Introduction

1.1 Research Background

Uterine adenomyosis is a common benign disease in gynecology. Most of them have secondary, progressive dysmenorrhea and menorrhagia as the main clinical manifestations, which seriously endanger the patient's physical and mental health. Most patients have the desire to retain the uterus, so the conservative treatment of drugs is dominant in the treatment of adenomyosis. However, the current various clinical medications of adenomyosis still have limitations and need to be improved, so it is imperative to expand the clinical drug use of adenomyosis [1]. Mifepristone is defined as a selective progesterone receptor modulator. Under certain conditions, it acts as an antiprogestin, inhibits endometrial proliferation, inhibits ovulation, and can cause reversible amenorrhea. Therefore, in recent years it has been widely used in uterine fibroids, endometriosis and other hormone-dependent gynecological diseases [2-5]. Adenomyosis is also associated with hormone-related diseases, especially with endometriosis closely. The protocols of clinical drug treatment are similar between them. At present, domestic research on mifepristone for the treatment of adenomyosis is relatively extensive. A large number of domestic reports have reported that short-term treatment of adenomyosis with low-dose mifepristone is safe and effective [6, 7]. During the treatment with mifepristone, most patients had amenorrhea, which significantly reduced dysmenorrhea and decreased menstrual flow. The effect on reducing the size of the uterus and lesions of patients with adenomyosis was significant [8, 9]. However, the majority of domestic literatures were small sample case-control studies or retrospective studies. There is no randomized, double-blind, controlled study. The clinical study of adenomyosis treated with Mifepristone is not found overseas. Therefore, the clinical evidence is insufficient, and high-quality randomized controlled trials are still needed.

This project intends to use multi-center, randomized, double-blind, controlled clinical trials to explore the effectiveness and safety of mifepristone (10 mg) for the treatment of symptomatic adenomyosis, which is a new clinical study of the old drug.

1.2 Research principle

Mifepristone is a selective progesterone receptor modulator. Foreign high-quality literatures have proved that it is effective for low-dose and short-term treatment of uterine fibroids and endometriosis. The purpose of this study was to confirm the effectiveness and safety of mifepristone (10 mg) for the treatment of symptomatic adenomyosis.

1.3 Benefit - Risk Analysis

In recent years, the clinical studies at home and abroad have proven that oral mifepristone treatment can reduce the volume of adenomyosis, achieve amenorrhea, relieve dysmenorrhea, improve anemia due to more bleeding, relieve clinical symptoms, and the treatment costs are relatively cheap. No significant adverse reactions were found in previous studies. Early studies showed that in early/middle follicular levels, GnRH-a low estrogen status was less likely to occur [10]. The psychological, life, economic, and other damages and adverse effects of the subjects were rarely caused. Only a few individuals experienced some known minor adverse side-effects after taking the drug. We define adverse events in detail in the study, and regularly check the various biochemical indicators of the patients and monitor endometrial thickness through pelvic ultrasound, etc. If the ultrasound results show endometrial thickness ≥12mm, endometrial biopsy is recommended to be performed. If the patient develops any discomfort during the study, or if there is a new change in the condition or any unexpected condition, regardless of whether it is related to the drug, the researchers will make judgments and medical treatment.

2. Research purposes

The main purpose of this study was that the visual analogue scale (VAS) was used as the main evaluation index to investigate whether daily oral mifepristone of 10 mg was superior to placebo in the treatment of symptoms of adenomyosis-associated pelvic pain.

The secondary purpose is to evaluate the pictorial blood loss assessment chart (PBAC), uterine volume, hemoglobin changes, and the safety of this dose (eg, adverse events, vagina bleeding, gynecological safety).

3. Researchers and other research participants

In this study, the main project leader is Professor Zhang Xinmei from Women's Hospital, School of Medicine Zhejiang University. The principal researchers must sign their names in the signature page of this program before each research center begins to recruit subjects. Similarly, before all program revision plans/integration plans become effective in the corresponding center, both name and date must be signed by the principal researchers. A complete list of all participating centers and their researchers, as well as all required signature files, will be kept in the research documents of the sponsor.

4. Research design

4.1 Overview of research design

This multicenter study is performed in 150 subjects who are diagnosed as adenomyosis by B-ultrasonography or magnetic resonance imaging (MRI) and had related symptoms (dysmenorrhea, with or without large doses).

Twelve weeks of randomization, allocation concealment, double-blind, placebo-controlled, parallel grouping. Subjects are randomly assigned to one of two treatment groups and received one of the following treatments: (1) Mifepristone tablets of 10mg, 1 tablet daily, oral; (2) Placebo, 1 tablet daily, oral.

4.2 The sketch map of research design

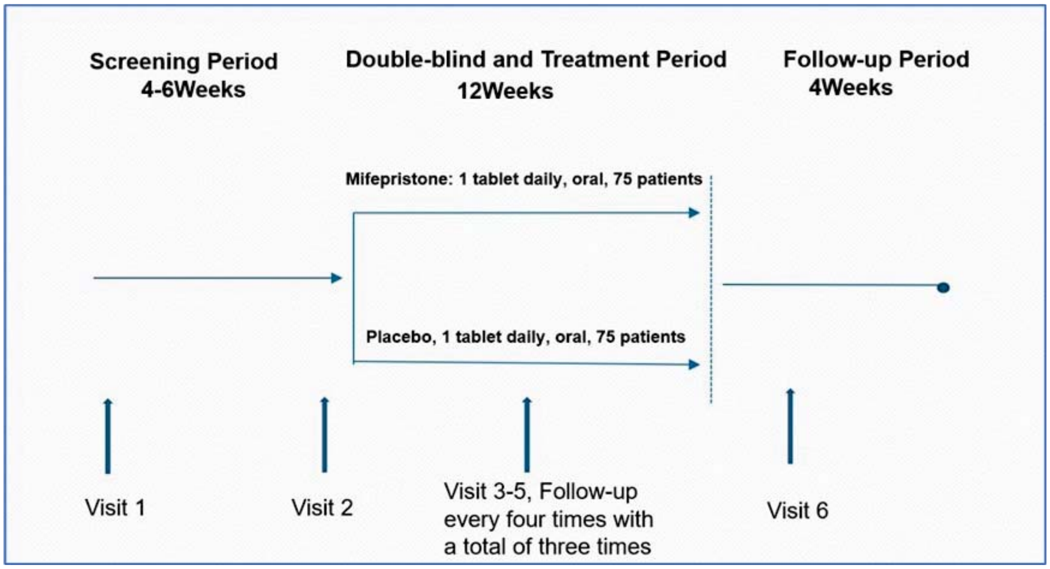

4.3 Rationality of research design

In order to avoid selection bias or bias by any other investigators' or subjects' understanding of the treatment used, the trial design was performed according to the principles of randomization, allocation concealment, double-blind, placebo control. The placebo control is necessary because of the more pronounced placebo effect observed in literature studies.

From an ethical point of view, placebo-controlled trials are considered acceptable because they allow the patients in the study to receive analgesics other than research drugs (indomethacin suppositories), which have been passed by the ethics Committee of Women's Hospital, School of Medicine Zhejiang University.

4.4 End of study

When the last participant of the research center in the entire study participates in the last visit, the entire study ends.

5. Study population

The study will include 150 patients from 16 hospitals in 12 cities in China.

5.1 Inclusion criteria

(1) Women had a diagnosis of adenomyosis by B-ultrasound or pelvic MRI examination and the volume of their uterus were less than those with 10 weeks of pregnancy

(2) Women with 10-cm visual analogue scale (VAS) of adenomyosis-associated pain > 0 point with or without menorrhagia (PBAC≥100 points)

(3) Women between 18 and 50 years of age who currently have no childbearing requirement

(4) Women who had normal cervical cytology test

(5) Women who agreed to choose a barrier method of contraception

(6) Women who agreed to adhere to the study requirements and signed the informed consent

(7) Women who were not postmenopausal

5.2 Exclusion criteria

(1) Patients with HB < 90G/L

(2) Patients with undiagnosed abnormal vaginal bleeding or endometrial lesions

(3) Patients who were preparing to give birth when taking the medication or within 6

months of stopping the medication

(4) Patients with malignant tumors (including the reproductive system and other systems);

(5) Patients with severe heart, liver, kidney disease and adrenocortical insufficiency

(6) Patients who had the results of follow-up laboratory test indicating abnormal clinical significance

(7) Patients who had a known allergy to mifepristone, or related compounds

(8) Patients with any other diseases or conditions that may cause the study drug to alter absorption, accumulate excessively, affect metabolism, or change the excretion pattern

(9) Patients with clinically significant depression within the current or most recent year

(10) Patients who regularly took analgesics due to other underlying diseases

(11) Patients used the drugs, such as Ketoconazole, itraconazole, erythromycin, rifampicin, corticosteroids (hydrocortisone, prednisone, dexamethasone, etc.), and some anticonvulsants (phenytoin, phenobarsbital, carbamazepine, etc.), griseofulvin, non-steroidal anti-inflammatory drugs (aspirin, acetaminophen, etc.) and couldn’t be stopped during the study

(12) Patients currently used any other hormone drugs, including: a. use of GnRH agonists within 6 months before the screening period; b. use of progestins or danazol and other long-acting hormones within 3 months before the screening period; c. use of oral contraceptive-like short-acting hormones within one month before the screening period;

(13) Patients who participated in other clinical trials within 3 months before the screening, or who were considered inappropriate to participate in the study by other investigators.

5.3 Quit research

5.3.1 Quit decided by the researchers

Refer to the fact that the subject who has already been selected cannot continue to conduct the test during the trial. The researcher decided that the case quit the trial.

(1) In the course of the study, if the subject's condition is aggravated (which is defined as: after at least 4 weeks of treatment, the formerly symptomatic subject has aggravated symptoms that severely affect life; or the patient with no previous symptoms has or develops new adenomyosis related symptoms that severely affect life; or abnormalities such as adenomyosis/ intima, and so on, found by the B-ultrasonic examination during the study period, and other researchers believe that the condition is aggravated), in order to protect the subject, let the subject complete the safety check and withdraw from the test and receive other effective treatments;

(2) In clinical trials, subjects have some comorbidities, complications, or special physiological changes that were not suitable for continued trials;

(3) Use of other treatments or drugs which are prohibited to be combined with each other, affects the judgment of effectiveness and safety.

(4) The subject has adverse events and serious adverse events (such as acute allergic reactions), who is not suitable to continue to undergo the trial;

(5) The use of the drug is less than 80% of the prescribed amount or more than 120% of the prescribed amount, but the clinical recovery standard is reached after the medication is not fully used, and the subject applies for the termination of the drug. With the consent of his/her responsible doctor, the remaining drugs can be returned and a separate description of compliance issues can be made;

5.3.2 Subjects withdrawing from the trial

According to the informed consent agreement, the subject has the right to withdraw from the trial midway, or if the subject does not withdraw from the trial explicitly, but no longer accepts the medication and detection to be lost, which also belongs to "quit" (or "drop off"). The reasons for their withdrawal should be understood as far as possible and recorded. For example: consciously poor efficacy; difficult to tolerate some of the adverse reactions; have something so that cannot continue to receive clinical research; economic factors; or lost due to unexplained reasons and so on. Regardless of the reason, the case record should be kept for the case of withdrawal from the trial.

5.4 Subject replacement

Subjects who do not complete the study under the trial protocol cannot be replaced.

5.5 Subject number

After signing the informed consent, subjects who meet all the inclusion criteria and do not violate exclusion criteria will receive a unique enrollment number.

6. Research drug and administration

6.1 Drugs to be given

(1) Mifepristone tablets, SFDA approval number: H20010633, Specification: 10mg/tablet, period of validity: 36 months, produced by China Resources Zizhu Pharmaceutical Co., Ltd.;

(2) Placebo, period of validity: 36 months, produced by China Resources Zizhu Pharmaceutical Co., Ltd.

6.2 Treatment Grouping

Twelve weeks of double-blind, randomization, placebo-controlled, parallel grouping. Subjects are randomized into 2 parallel dose groups and receive 10 mg of mifepristone tablets or placebo, one tablet daily, orally.

In the grouping process, the principle of randomization, allocation concealment, and double-blindness was used. The subject will receive a unique enrollment number, and the drug will also be assigned a unique number. The drug number will be pre-labeled on the package containing the study drug. The subject number and drug number are all unique and corresponding, and are handled by a special person and keep confidential for the clinician and the subject. This person does not participate in any part of the study of the trial. Neither the subject nor the investigator knows the characteristics of the assigned study drug. After the double-blind part is over, all subjects need to receive the same treatment.

6.3 The package of trial drug

Drugs have the unified label format, of which the contents include: drug number, clinical research drug name (for clinical research use only), indications, usage and dosage, course of treatment, storage conditions, lot number, expiration date, drug supply agency, etc.

6.4 Distribution, preservation and recycle of research drug

The trial drug cupboard is locked, and sealed tightly, protected from light. Each research organization designates a research drug manager. The researchers screen qualified subjects, sign

informed consent, and write medical records of research, and register them in the "Trial Drug Management Registration Record Form". The subjects are also given drug recycle instructions to inform the subjects that they would need to put the remaining drugs into the drug packaging box after the end of clinical drug use. At the time of follow-up, the remaining drugs are returned to the research drug manager and stored separately. At the end of clinical study, the remaining drugs will be returned to the sponsor or destroyed.

6.5 Administration of the research drug

6.5.1 Methods of administration

Subjects are given the research drug orally once a day, starting on the third day of menstruation, taking one tablet on an empty stomach or 2 hours after eating, and fasting for 2 hours after taking the drug. It is best to take the medicine at the same time every day and take it with warm water. Patients must continue to take tablets. If a pack of medication is used up, the next pack must be started without interruption.

6.5.2 Handling of forgetting medication

If the patient forgets to take medication, or has vomiting and/or diarrhea (if it occurs within 3 to 4 hours after taking the tablets), the effectiveness of the drug may be reduced. If the subject forgets to take one tablet or more tablets of drug, he/she must take only one tablet of drug when remember, and continue to take medication on the following day at regular time. Tablets that have not been absorbed due to vomiting or diarrhea must be replaced with the same tablets.

6.5.3 Special Instructions

Subjects are allowed to follow a regular diet. However, during the study period, the diet of subjects must not include grapefruit and grapefruit juice because these foods contain cytochrome P450 isoenzyme 3A4 (CYP3A4), which is involved in the biosynthesis and/or degradation of certain endogenous compounds such as steroid hormones, cholesterol and fatty acids and can participate in the metabolism of mifepristone. Subjects are allowed to ingest alcohol but not to exceed 30 g daily (equivalent to 500-750 mL beer, 250 mL wine, or 75 mL spirit).

6.5.4 The necessity of contraceptive measures

After a one-month treatment, mifepristone tablets can result in anovulation. However, placebo does not result in this condition. Therefore, to maintain blindness, all subjects who may become pregnant need to use non-hormonal methods (barriers to contraception, such as condoms) during the study.

6.6 Blind method

The study is a double-blind study. Mifepristone tablets have the same appearance (size, shape, and color) as placebo tablets. In different treatment groups, the packaging of the research drug is the same, so that the investigator or subject or sponsor cannot know the assigned treatment.

The treatment of the double-blind part remains blind until the part of the database is locked. In accordance with applicable regulations, in case of suspected, unintended, serious adverse reactions, if consideration is given to blinded treatment, the subject's treatment number is usually unmasked before being reported to health authorities, ethics committees and researchers.

6.6.1 Break code by researchers in an emergency

In the event of an emergency, the researchers can break the code for the individual case. In exceptional cases, the code may be broken urgently, for example, ensuring that the patient's treatment allocation can be checked at any time and under all conditions in the research center. However, the preferred method is not to use emergency broken envelopes.

If breaking the code, the subject will no longer be eligible to continue to participate in the study. If the code is broken due to a medical emergency, the researcher needs to immediately inform the clinical research leader of sponsor.

If the reason of breaking code is a serious adverse event, the researcher must record the actual treatment that the subject received in the list of serious adverse events.

However, the occurrence of serious adverse events does not mean that the treatment given to the subject needs to be broken. Researchers can break the randomized code only if treatment information is important for acute treatment strategies. If the code of subject needs to be broken, it must make every effort to get in touch with the clinical research leader of sponsor before breaking the randomized code. If this cannot be achieved, the sponsor must be contacted within 24 hours after breaking the randomized code. The date, time, and reason of breaking code must be recorded.

6.6.2 Break code by sponsor in an emergency

In accordance with applicable regulations, in case of suspected, unintended, serious adverse reactions, and if consideration is given to blinded treatment, the treatment code of subject is usually broken before being reported to medical institutions, ethics committees, and researchers.

6.7 Post-study treatment

At the end of the study, subjects will receive standard therapy/treatment according to the researcher's decision.

6.8 Combining medication and treatment

(1) In addition to the trial drug, it is forbidden to use other Chinese and Western drugs treating this disease that are the same or similar to the functional indications of mifepristone tablets in the observation period, and other drugs and treatment methods that affect the evaluation of the efficacy of this trial, including other contraceptive drugs (such as progestin contraceptives, estrogen-progestin contraceptives, etc.); and drugs that interact with mifepristone (such as ketoconazole, itraconazole, erythromycin, etc., and rifampicin, corticosteroids, and some anticonvulsants, griseofulvin, non-steroidal anti-inflammatory drugs, and so on). Please refer to the instructions for drug interactions for details. It is forbidden to take painkillers and hemoglobin drugs privately without the consent of a doctor.

(2) During the study period, the endometrium thickness of the B-ultrasonic examination should be recorded in the eCRF. If the researcher suspects endometrial hyperplasia, hysteroscopy or curettage examinations can be performed if necessary, and with reference to clinical routine treatment, the study medication can be discontinued or continue to be used.

(3) During the observation period, the name, usage and dosage, and time of taking of drugs or other treatments that must be taken for other comorbid diseases, must be recorded in the electronic case report form.

7. Research steps and variables

7.1 Research steps

7.1.1 The flow chart

Table 7-1 The flow chart of clinical research

**Interview**	**Screening period**	**Baseline period**	**Double-blind treatment period**			**Follow-up period**
Number of interview	First	Second	Third	Fourth	Fifth	Sixth

**Interview**	**Screening period**	**Baseline period**	**Double-blind treatment period**			**Follow-up period**
Time point of interview	-42~0 day	0~3 day	The fourth week ±4 days	The eighth week ±4 days	The twelfth week ±4 days	The sixteenth week ±4 days
**Basic history collection**						
Signing informed consent	√					
Eligibility screening	√					
Filling in general information	√					
Vital Signs/Anthropometrics	√		√		√	
Gynecological examination	√				√	
**The follow-up of auxiliary examination**						
Blood routine	√		√		√	√
Coagulation function	√					
Liver and kidney function and blood	√		√		√	

**Interview**	**Screening period**	**Baseline period**	**Double-blind treatment period**			**Follow-up period**
lipids						
Three sex hormone tests	√		√		√	√
CA_{125}	√				√	√
Blood/Urine HCG	√		√		√	
Pelvic B ultrasound	√		√		√	√
Pelvic MRI	√(Non-essential)				√(Non-essential)	
Breast B ultrasound	√					
Leucorrhea routine	√					
ECG	√					
**Questionnaire follow-up**						
VAS	√		√	√	√	√
PBAC	√		√	√	√	√
**Other work**						
Endometrial biopsy	√(when necessary)				√(when necessary)	
Dispensing trial drugs		√	√	√		

Interview	Screening period	Baseline period	Double-blind treatment period			Follow-up period
Recording adverse events and drug combination			√	√	√	√
Compliance judgment (medication and follow-up)			√	√	√	

7.1.2 Assessment schedule

7.1.2.1 Interview 1- Screening period (-42 to 0 day)

The following data is collected and the following assessments are performed 4 to 6 weeks before starting research drug treatment:

(1) Signing informed consent (before conducting any other study-related assessment described below)

(2) Eligibility screening (according to the inclusion criteria and exclusion criteria)

(3) Filling in general information

(4) Vital signs/Anthropometrics (blood pressure, heart rate, height, weight, gynecological examination)

(5) Completion of related auxiliary examinations (blood routine, coagulation function, blood/urine human chorionic gonadotropin, CA125, liver and kidney function, blood lipids, three sex hormone tests, pelvic ultrasonography (uterine length, width, thickness), pelvic MRI (non-essential), breast B ultrasound, ECG, leucorrhea routine)

(6) Distributing questionnaires (VAS, PBAC)

(7) Endometrial biopsy (when necessary)

7.1.2.2 Interview 2- Baseline period (0 to 3 day)

(1) Improving auxiliary examinations

(2) Randomization group and dispensing study drugs

7.1.2.3 Interview 3- Taking medicine for 1 month (The fourth week ±4 days)

(1) Measurement of vital signs (blood pressure, heart rate)

(2) Completion of related auxiliary examinations (blood routine, liver and kidney function, blood lipids, three sex hormone tests, blood/urine human chorionic gonadotropin, pelvic ultrasonography (uterine length, width, thickness))

(3) Recording adverse events and drug combination

(4) Compliance judgment

(5) Distributing questionnaires (VAS, PBAC)

(6) Dispensing study drugs

7.1.2.4 Interview 4- Taking medicine for 2 months (The eighth week ±4 days)

(1) Recording adverse events and drug combination

(2) Compliance judgment

(3) Distributing questionnaires (VAS, PBAC)

(4) Dispensing study drugs

7.1.2.5 Interview 5- Taking medicine for 3 months (The twelfth week ±4 days)

(1) Vital signs/Anthropometrics (blood pressure, heart rate, height, weight, gynecological examination)

(2) Completion of related auxiliary examinations (blood routine, liver and kidney function, blood lipids, three sex hormone tests, blood/urine human chorionic gonadotropin, CA_{125}, pelvic ultrasonography (uterine length, width, thickness), pelvic MRI (non-essential))

(3) Counting and recycling drugs

(4) Recording adverse events and drug combination

(5) Compliance judgment

(6) Distributing questionnaires (VAS, PBAC)

(7) The result of ultrasound shows endometrial thickness ≥12mm or suspected abnormal endometrium, and endometrial biopsy is recommended.

7.1.2.6 Interview 6- Drug withdrawal for 1 month (The sixteenth week ±4 days)

(1) Completion of related auxiliary examinations (blood routine, CA_{125}, three sex hormone tests, pelvic ultrasonography)

(2) Distributing questionnaires (VAS, PBAC)

(3) Recording adverse events and drug combination

7.2 Effectiveness

7.2.1 Visual Analogue Scale (VAS)

7.2.2 Pictorial blood loss assessment chart (PBAC)

Effectiveness variable - scored by PBAC - menstrual flow status prior to the assessment time point. At the same time, the uterine bleeding in the first 4 weeks was evaluated.

7.2.3 Changes in uterine volume

Effectiveness variable - uterus length, width, and thickness measured by B-ultrasound, volume (cm3) = length x width x thickness x 0.5236 - changes of the uterine volume were compared within group before and after treatment (0, 12, 24 weeks and follow-up period).

7.2.4 Anemia improvement

The changes of anemia related indicators (red blood cell count, hemoglobin) relative to baseline of each interview in each group are described and compared within group before and after treatment (0, 4, 12, 16weeks).

8. Safety indicators

The safety assessment will include the monitoring and recording of all adverse events (AEs) and serious adverse events (SAEs). Routine blood tests, blood biochemistry and vital signs as well as physical examination, ECG, and endometrium test will be performed regularly according to routine medical procedures.

9. Quality control and guarantee

In order to further ensure the quality of clinical trials, a multi-center trial coordination committee is established to appoint the general director of clinical research and serve as the research coordinator among the clinical trial centers. The persons in charge of the research units and the persons in charge of the sponsors are members of the coordination committee. The Coordinating Committee is responsible for the implementation of the entire test and resolves issues related to the trial;

The sponsor will appoint the principal investigator to ensure that the rights of the subjects in the clinical trials are safeguarded, the data of the trial records and reports are accurate, complete and correct, and to supervise the implementation of clinical trial programs, the clinical trial management regulations and relevant laws and conduct regular on-site supervision visits to each center;

For eCRF forms that do not meet the program, have the right to ask the researcher who participates in clinical trials to re-enter them, but they must follow the authenticity of the information.

Through pre-clinical training, researchers are fully aware of and understand the clinical trial program and the specific contents of each indicator. For the objective indicators specified, inspections should be conducted according to the timing and methods prescribed by the program. Attention should be paid to the observation of adverse reactions or unexpected toxic side effects, and follow-up observations are performed.

10. The methods of observation, record and report of adverse events

10.1 Adverse events

The term of adverse events encompasses any clinical syndrome, symptom and syndrome that occurs in the subject and affects the health of the subject, or the appearance or worsening of a disease during the clinical study. The term also includes other clinically relevant conditions that occur in the laboratory and during the testing process, for example, unplanned measures of diagnosis and treatment, or resulting in withdrawal from the trial. Adverse events may be: new disease; worsening of symptoms or signs of treatment status, or worsening of the accompanying disease; not related to participating in the trial; a combination of one or more factors. Therefore, the term "adverse event" does not mean a causal relationship with the trial drug.

10.2 Serious adverse events

The following adverse events occur at any dose of trial drug or at any time during the observation period, including: leading to death; immediate life-threatening; need of hospitalization or prolonged hospital stay; disability; leading to congenital malformations; important medical significance (referring to those that do not immediately endanger life or cause death or need hospitalization, but may harm the patient or need to take steps to prevent one of the consequences defined above) requiring medical treatment to prevent permanent injury or damage.

10.3 Medical side effects

Harmful rather than expected reactions which has a causal relationship with the drug use is generated during the normal application of the drug at the prescribed dose. In clinical trials of a new drug or new use of a drug, when its therapeutic dose has not been determined, all adverse but not expected reactions that have a causal relationship with drug use should also be regarded as adverse drug reactions.

10.4 Severity of adverse reactions

Mild: can be tolerated by the patient, does not affect continued treatment, does not require special treatment, and has no effect on the rehabilitation of the patient.

Moderate: cannot be tolerated by the patient, and needs to be discontinued or given special treatment, and has a direct effect on the rehabilitation of the patient.

Severe: endangers the patient's life, disability or death, and immediate withdrawal or urgent treatment is required.

10.5 Judgment of causality between adverse reactions and drugs

10.5.1 Related indicators of causality judgment

(1) Whether there is a reasonable sequential relationship between the time of starting drug use and the time when the suspected adverse reaction occurs.

(2) Whether the suspicious adverse reaction meets the known adverse reaction type of the drug.

(3) Whether the suspected adverse reactions can be explained by the patient's pathological condition, drug combination, combination therapy, and previously used therapy.

(4) Whether the suspicious adverse reactions can be reduced or disappeared by withdrawal or reducing the dose.

(5) Whether or not the same adverse drug reactions occur again after receiving the same drugs

again.

10.5.2 Causality judgment

Based on the above five indicators, the analysis of causality is divided into 5 levels of affirmative relevance, probable relevance, possible relevance, suspicious relevance, and impossible relevance.

Causality judgment of adverse reactions

	**Affirmative relevance**	**Probable relevance**	**Possible relevance**	**Suspicious relevance**	**Impossible relevance**
A reasonable time sequence with the trial drug	+	+	+	+	-
Known types of adverse reactions of drug	+	+	+	-	-
Relief or disappear after drug withdrawal	+	+	±	±	-
Adverse reactions return or disappear after re-administration	+	?	?	?	-
Cannot be explained by subject's disease	+	+	-	±	-

Note: (1) "+" means affirmation; "-" means disavowal; "-" means difficulty in affirming or denying; "?"means that the situation is not clear. (2) Affirmative relevance, probable relevance can be considered as adverse reactions caused by drugs.

10.6 Adverse event handling

10.6.1 Record and report

The researcher should explain to the patient and ask the patient to truthfully reflect the changes in condition after medication. Physicians avoid suggestive questions.

While observing the efficacy, pay attention to observe adverse reactions or unexpected toxic side effects (including symptoms, signs, laboratory inspection). No matter whether the adverse reactions or adverse events are related to the trial drug, they should be recorded in detail, including the time of appearance of adverse reactions, symptoms, signs, degree, duration, laboratory test indicators, treatment methods and results, the course and follow-up time and so on. The details of the drug combination are recorded in order to analyze the correlation between adverse reactions and the trial drugs.

Records should be signed and dated. If there are serious adverse events during the trial, the researcher must report to the local provincial food and drug supervision and administration

department, the State Food and Drug Administration, the sponsor, the clinical research responsible unit and the medical ethics committee within 24 hours. Researchers should sign and date the report. It should be recorded when, in what way and to whom serious adverse events were reported in the source data. The sponsor should immediately notify the participating units and ensure to meet reporting procedures required by all laws and regulations.

10.6.2 The treatment of the subjects

When an adverse reaction is found, the researcher can take the necessary measures according to the condition. For example: adjusting the dosage of the trial drug, temporarily discontinuing the drug, permanently discontinuing the drug, taking the accompanying drug, and so on. In the event of a serious adverse event, the unit that undertakes the clinical trial must immediately take necessary measures to protect the safety of the subject. All adverse events should be tracked and the details of the treatment process and results should be recorded until they are properly resolved or the condition is stable. If abnormalities are detected, they should be traced back to normal.

Hospital, outpatient, home visits, telephone, communications and other forms of follow-up methods can be selected according to the severity of adverse reactions.

11. Study medical records, eCRF filling and transfer

Each case in group must complete the medical record and electronic case report form, which filled in by the researcher and inspected by the chief research officer before data entry and management.

12. The relevant requirements of observation, record and summary

(1) Cases involved in this clinical trial are required to sign patient informed consent.

(2) Carefully observe, record and fill in the electronic case report form according to the program and observation form. The electronic case report form must not be absent.

(3) Carefully record the subject's medication and record in detail the reasons for not using the medication as prescribed.

(4) The research medical record and electronic case report form shall not be arbitrarily modified. If there is a need for change, the researcher shall sign and date at the amendment department (the electronic case report form will be automatically recorded).

(5) The study medical record of outpatient cases should be affixed with the original laboratory examination report form, while the study medical record of hospitalized case should be affixed with a copy of the laboratory examination report form.

(6) After the observation is over, all electronic case report forms are reviewed by the leaders of each participating organization and uploaded to the EDC. The original medical record is preserved by the recode department of each participating organization.

(7) Clinical trial statistics organization perform the statistical analysis with all cases of each trial center, and provided the statistical results to the participating organizations to write the “Clinical Trial Summary”. Finally, the responsible institution should complete the "Clinical Trial Summary Report”.

13. Medical ethics requirements and protection of subjects' rights and interests

This clinical trial will follow the Helsinki Declaration (Fortaleza, Brazil, the 64th World Medical Association Joint Conference, 2013) and relevant drug clinical trials research norms and regulations in China. The clinical trial plan must be approved by the Medical Ethics Committee of the responsible research institution before implementation. The investigator must provide the subject with detailed information about the clinical trial and sign the patient's informed consent prior to the trial. If clinical trial need to modify the original plan, they must be reported to the Medical Ethics Committee and approved before implementation. Subjects can receive timely medical treatment when adverse reactions occur during the trial.

14. Subject's medication compliance

In this clinical trial, attention should be paid to the subject's compliance with the use of medication. Patients should be fully aware of the importance of on-time medication and strictly follow the prescribed medication. The reasons for not using the prescribed drugs should be recorded in detail.

15. Data management and statistical analysis

15.1 Monitoring of Data Records

The leader of the responsible institution shall periodically visit each trial center to check the subject's informed consent and screening situation during the trial process, as well as confirm that all eCRFs are filled in correctly and consistent with the original data. Besides, the investigator shall also confirm that all errors or omissions have been corrected or indicated and then signed and dated. Furthermore, the situation of each subject's dose change, treatment change, combined medications, intercurrent illness, loss of follow-up, examination omissions, etc. should be exactly confirmed and recorded and the verification of the subject's withdrawal must be described in the eCRF.

The leader shall also confirm that all adverse events have been recorded and especially serious adverse events have been recorded and reported within the specified time. What's more, the leader should verify whether the trial drug has been supplied, stored, distributed, and withdrawn in accordance with relevant regulations and records has been made accordingly.

When verifying the original medical records, it should be noted that the records should be timely, accurate, truthful, standardized, and complete. In particular: (1) Whether the eligible subjects meet the inclusion and exclusion criteria; (2) Whether the names, dosages, and usages of the trial drugs are recorded on the original medical records and whether the prohibited drugs prescribed in the clinical trial program are used; (3) Whether the original medical record observation, examination items and records are consistent with the eCRF. (4) Whether the degree of severity of illness, efficacy determination and safety evaluation are correct. (5) Whether the name, address, telephone number, medical record number and admission number of the subject is filled in exactly. (6) Carefully record the patient's medication situation, make detailed records of the patient's compliance, and especially make a detailed record of the situation of all use, sometimes use, and half of the unused. (7) Data within the normal range should be recorded, while data that is significantly higher or outside the clinically acceptable range should be verified and recoded by the physician participating in the clinical trial. (8) Clinical observation forms should always be recorded using a pen. Each page needs to be signed and dated.

15.2 Data entry and modification

EDC software is used to compile data entry procedures for data entry and management by data administrators which jointly designated by the sponsor and the statistical organizations. In order to ensure the accuracy of the data, two data entry staff should be independently entered twice and compared.

For questions in the electronic case report form, the data administrator will generate a question answering form and issue an inquiry to the researcher through the clinical monitor. The researcher should answer and return as soon as possible. The data administrator will modify, confirm and entry the data according to the researcher's answer. If necessary, the data administrator can re-generate a question answering form.

15.3 Data locking

When all electronic case report forms (eCRF) are entered into the EDC database in duplicate, and after questioning and verification, the sponsors and statisticians lock the data. Hence, the locked data files cannot be modified anymore.

15.4 Selection of statistical analysis data

Full Analysis Set (FAS): According to the basic principles of intentionality analysis (ITT), statistical analysis is performed on all randomized cases and patients without any valid data at baselined will be excluded. For the uncompleted clinical trial observations data, last-observation-carry-forward is conducted at the end of the trial. The number of subjects whose efficacy will be evaluated at endpoint is the same as at the beginning of the trial. Per Protocol Set (PPS): All data from those well compliant subjects (taking 80% to 120% of the applied trial drug dose), which compliant with the protocol and have not taking the prohibited drug during the trial, as well as have VAS assessed at baseline, and have at least one follow-up VAS record. Safety Set (SS): All subjects who were randomized and have taken at least once clinical trial drug and have at least once safety evaluation should be evaluated for safety.

15.5 Statistical analysis method

(1) General principles

SAS is used for statistical analysis.

Two-sided tests are used in all statistical tests with an Alpha level of 0.05.

The mean, standard deviation, median, minimum, maximum, and interquartile range will be calculated in the description of quantitative indicators.

Various types of cases and percentages are used in the description of the classification index.

(2) Case characteristics

Enrollment and completion: Summarize the number of cases enrolled and completed by each center, and list the unfinished cases and cases that do not enter each population.

(3) General information and baseline characteristics

The baseline is defined as Interview 1 (Day 0-3), and the SS is evaluated at baseline.

Baseline descriptions are made on the age, sex, allergy history, medication history, history of illness, and laboratory tests for each group of population.

(4) Analysis of safety indicators

The SS population is used for analysis.

Describe the number of occurrences of adverse events and adverse reactions of the total and each group and give the corresponding list in the detailed account.

Compared with the laboratory tests before the treatment, the situations after the treatment of each interview are described in the form of the crosstabs, and the abnormalities that are significant after the treatment are listed.

The vital signs indicators of each interview relative to the baseline during interviews and after treatment are described, and t-test or non-parametric test is used for intragroup comparison.

(5) Analysis of efficacy indicators

FAS and PP populations are used for analysis.

Baseline: Baseline description of each efficacy indicator.

Primary effectiveness variable

The primary effectiveness variable is assessment of adenomyosis-associated pain by VAS at week 12 (at the end of the double-blind portion) and compared with baseline period (interview 2). Using the covariance analysis (ANCOVA), baseline VAS scores are used as covariates to compare the differences in the VAS scores at the 12th week. At the same time, comparisons were made during each interview (interview 2-5). For the missing data, the method of Last Observation Carried Forward (LOCF) will be used. Further details of the method can be found in the statistical analysis plan.

Secondary effectiveness variable

Pictorial blood loss assessment chart (PBAC), uterine volume and hemoglobin changes, and safety (e.g., adverse events, vaginal bleeding, gynecological safety).

The detailed analysis of secondary effectiveness variable is seen in the statistical analysis plan.

Safety variable

Descriptive analysis of safety variables will be performed. The relative change in BMD (expressed as a percentage) at 12 weeks and at the end of treatment compared to baseline will be presented as descriptive statistics. In addition, 95% confidence intervals will be provided for each treatment

group.

(1) Medication compliance

Medication compliance is described by measurement data.
Compliance is segmented by <80%, 80-120%, and >120%, and is described by ranked data.

Determination of sample size

In correlational studies, mifepristone treatment of adenomyosis reduced the VAS score from 8.49±1.83 before treatment to 2.01±0.87. The average value before and after treatment decreased by 6.48. Although there are many related literatures about placebo, they are quite different. Based on the principle of conservation, we make the following assumptions:

- The VAS score after 12 weeks of treatment in the mifepristone group was approximately 2.01;
- The VAS score after 12 weeks of treatment in the placebo group was approximately 3.25;
- The standard deviation of VAS scores between two groups after treatment was similar (about 2.31).

Using a margin of error of α=0.05 and a potency of 0.9 (β=0.1) in a two-sided test, 60 subjects need to be evaluated in each treatment group, which can demonstrate that mifepristone is superior to placebo. After considering loss of follow-up, violations of research protocols, compliance and other factors, each group needs to add 25% of the subjects, thereby ensuring that at least 60 subjects in each group complete the study. Therefore, at least 75 subjects should be selected for each treatment group.

15.6 The monitoring system

The person in charge of the sponsor conducts a systematic examination of the clinical trials and monitors the trials in accordance with the GCP principle, in order to ensure that the trial program is conducted in accordance with regulations and that the data recorded in the case reporting forms are the same as the original data.

16. The management of clinical trial drug

16.1 Trial drug packaging

Each hospital distributes the corresponding numbered drugs, and the research drugs are uniformly stored and managed by the trial institution. Each center screens patients, and the qualified ones are

selected, and the trial drug distributor distributes the drugs according to the order of the number of trial drugs for each subject (note: those who meet the inclusion criterion may be given the drugs). The trial drug distributor shall promptly fill in the "Trial Drug Management Registration Record" and the remaining drugs shall be recycled and then destroyed. Each package comes with a drug label, which contains the statutory contents such as approval number, drug number, drug name, quantity, major functions, usage, storage conditions, and drug supply institution.

16.2 Trial drug distribution

After China Resources Zizhu Pharmaceutical Co., Ltd. uniformly prepares and packages the trial drugs, the trial drugs are distributed according to the required amount to the Women's Hospital, School of Medicine Zhejiang University, and managed by special personnel and distributed to each trial center.

Each trial center is uniformly accepted by special personnel to manage trial drugs and handle the handover procedures.

According to the drug number, the drugs will be sent to the subjects by special personnel according to the number of the subject's visit in the order from the smallest to the largest in each trial center. Each center shall register the clinical release status of the drug, and at the end of the trial, the registration record of trial drugs shall be submitted to the sponsor for examination by the drug regulatory department.

16.3 Trial drug inventory

Trial drug supply: The applicant provides trial drugs (certifications for trial drugs) in accordance with the requirements of clinical trials, and the researcher establishes the record cards of clinical trial drug administration to check whether the quantity, quality, and the drug code of the trial drug are correct.

Trial drug distribution: In the process of trial drug distribution, special personnel will check the drug, record the usage, and ensure that the drug is properly dispensed in accordance with the principle of sequence of visits.

Trial drug recovery: After the end of the trial, special personnel will perform recovery and destruction of the remaining trial drugs.

16.4 Trial drug preservation

Trial drugs should be uniformly managed and preserved, and be handed out by special personnel. Select appropriate storage locations, ensure scientific storage conditions, and regularly check the quality and storage environment of trial drugs.

17. Modification of the program

After the program is approved by the Ethical Committee, if any changes are made, a "program modification explanation" must be written and signed by the principal researcher, and it must be approved by the ethics committee before implementation. After the program is revised, it must be accepted and signed by the sponsor; anyone who participates in the trial must not violate the program.

18. Summary and preservation of data

18.1 Data summary

After the completion of the clinical trial, the statistical institution will process the data according to the statistical requirements. Each clinical trial participant shall write a clinical trial summary form according to the unified requirements. The clinical trial responsible institution shall summarize the trial summary forms of each trial participant and write a clinical trial summary report. Based on this, an overall assessment of the clinical efficacy and safety of the drug is made.

18.2 Data preservation

Researcher saves all research data, including confirmation of all participants (can effectively check different records, such as hospital records), all original signed informed consent of patients, all study records, electronic case report forms, detailed records of drug distribution, etc. The researchers should keep the clinical trial data for five years after the termination of the clinical trial.

19. References

[1] Pontis A, D'Alterio MN, Pirarba S, de Angelis C, Tinelli R, Angioni S. Adenomyosis: a systematic review of medical treatment. Gynecol Endocrinol. 2016. 32(9): 696-700.

[2] Eisinger SH, Meldrum S, Fiscella K, le RHD, Guzick DS. Low-dose mifepristone for uterine leiomyomata. Obstet Gynecol. 2003. 101(2): 243-50.

[3] Chwalisz K, Perez MC, Demanno D, Winkel C, Schubert G, Elger W. Selective progesterone receptor modulator development and use in the treatment of leiomyomata and endometriosis. Endocr Rev. 2005. 26(3): 423-38.

[4] Williams AR, Critchley HO, Osei J, et al. The effects of the selective progesterone receptor modulator asoprisnil on the morphology of uterine tissues after 3 months treatment in patients with symptomatic

uterine leiomyomata. Hum Reprod. 2007. 22(6): 1696-704.

[5] Donnez J, Tatarchuk TF, Bouchard P, et al. Ulipristal acetate versus placebo for fibroid treatment before surgery. N Engl J Med. 2012. 366(5): 409-20.

[6] Chen S, Sun W, Zhu L, et al. Comparative analysis of the therapeutic effects of mifepristone with different low doses for the treatment of adenomyosis women with dysmenorrhea. Progress in Obstetrics and Gynecology. 2016. 25(12): 881-885.

[7] Chen G, Jiang Q. Comparative study of different doses of mifepristone in patients with uterine adenomyosis. Anhui Medical and Pharmaceutical Journal. 2012. 16(11): 1677-1678.

[8] Li L. Effect observation on different doses of mifepristone in the treatment of adenomyosis. China Practical Medical. 2017. 12(32): 95-97.

[9] Lv J, Sui L, Liu H. Study on Mifepristone treatment in the Patients with adenomgosis. Chinese Journal of Family Planning. 2002. (11): 686-688.

[10] Spitz IM, Grunberg SM, Chabbert-Buffet N, Lindenberg T, Gelber H, Sitruk-Ware R. Management of patients receiving long-term treatment with mifepristone. Fertil Steril. 2005. 84(6): 1719-26.
