# Supplementary material for: Effect of Mifepristone vs Placebo for Treatment of Adenomyosis With Pain Symptoms: A Randomized Clinical Trial
Source: JAMA Netw Open. 2023 Jun 12;6(6):e2317860. doi: 10.1001/jamanetworkopen.2023.17860 (PMC10261993; doi:10.1001/jamanetworkopen.2023.17860)
Supplement: Supplement 2. — eAppendix 1. Study Sites and Principal Investigators eAppendix 2. Inclusion Criteria and Exclusion Criteria eAppendix 3. Method of Sample Size Calculation eTable 1. Change in VAS After Mifepristone Treatment eTable 2. Total Effective Rate for Dysmenorrhea After Mifepristone Treatment eTable 3. Complete Remission Rate for Dysmenorrhea After Mifepristone Treatment eTable 4. Change in PBAC for Heavy Menstrual Bleeding After Mifepristone Treatment eTable 5. Total Effective Rate for Heavy Menstrual Bleeding After Mifepristone Treatment eTable 6. Complete Remission Rate for Heavy Menstrual Bleeding After Treatment eTable 7. Changes From Baseline in Hb, CA125, PLT and Uterine Volume After Mifepristone Treatment eTable 8. Relevant Laboratory Evaluations Changed From Normal to Abnormal After Mifepristone Treatment eTable 9. Changes in Liver Function in the 2 Groups During Treatment eTable 10. Comparison of the Changes of Endometrial Thickness Between the 2 Groups After 4 Weeks of Treatment eTable 11. Comparison of the Change of Endometrial Thickness Between the 2 Groups After 12 Weeks of Treatment eTable 12. Comparison of the Endometrial Thickness Between the 2 Groups During the Treatment eFigure 1. Flowchart of Study Enrollment eFigure 2. Levels of FSH, LH and E2 Between the 2 Groups at Different Periods of Treatment eFigure 3. Changes in Endometrial Thickness and BMI Between the 2 Groups at Different Periods of Treatment [file jamanetwopen-e2317860-s002.pdf]

Supplemental Online Content

Che X, Wang J, Sun W, et al. Effect of mifepristone vs placebo for treatment of adenomyosis with pain symptoms: a randomized clinical trial. *JAMA Netw Open*. 2023;6(6):e2317860. doi:10.1001/jamanetworkopen.2023.17860

eAppendix 1. Study Sites and Principal Investigators
eAppendix 2. Inclusion Criteria and Exclusion Criteria
eAppendix 3. Method of Sample Size Calculation
eTable 1. Change in VAS After Mifepristone Treatment
eTable 2. Total Effective Rate for Dysmenorrhea After Mifepristone Treatment
eTable 3. Complete Remission Rate for Dysmenorrhea After Mifepristone Treatment
eTable 4. Change in PBAC for Heavy Menstrual Bleeding After Mifepristone Treatment
eTable 5. Total Effective Rate for Heavy Menstrual Bleeding After Mifepristone Treatment
eTable 6. Complete Remission Rate for Heavy Menstrual Bleeding After Treatment
eTable 7. Changes From Baseline in Hb, CA125, PLT and Uterine Volume After Mifepristone Treatment
eTable 8. Relevant Laboratory Evaluations Changed From Normal to Abnormal After Mifepristone Treatment
eTable 9. Changes in Liver Function in the 2 Groups During Treatment
eTable 10. Comparison of the Changes of Endometrial Thickness Between the 2 Groups After 4 Weeks of Treatment
eTable 11. Comparison of the Change of Endometrial Thickness Between the 2 Groups After 12 Weeks of Treatment
eTable 12. Comparison of the Endometrial Thickness Between the 2 Groups During the Treatment
eFigure 1. Flowchart of Study Enrollment
eFigure 2. Levels of FSH, LH and E2 Between the 2 Groups at Different Periods of Treatment
eFigure 3. Changes in Endometrial Thickness and BMI Between the 2 Groups at Different Periods of Treatment

This supplemental material has been provided by the authors to give readers additional information about their work.

© 2023 Che X et al. *JAMA Network Open.*

eAppendix 1. Study Sites and Principal Investigators

Names of hospitals participating in clinical trials (No. of the center)	Principal investigators of each center
Women's Hospital, School of Medicine Zhejiang University (01)	Xinmei Zhang
Anhui Province Cancer Hospital (02)	Weidong Zhao
Huzhou Maternity and Child Care Hospital (03)	Dong Jie
Jiaxing Maternity and Child Health Care Hospital (04)	Weili Zhu
Jinhua Municipal Central Hospital (05)	Hu Min
The Central Hospital of Lishui City (06)	Hongyou Zhou
The Affiliated Hospital of Medical School of Ningbo University (07)	Zhang Jing
Ningbo Maternity and Child Care Hospital (08)	Qiming Wang
The People's Hospital of Quzhou City (09)	Jingui Xu
Shaoxing Women and Children's Hospital (10)	Yungen Wang
Shaoxing People's Hospital (11)	Haigang Ding
The First Affiliated Hospital of Wenzhou Medical University (12)	Feiyun Zheng
The Second Affiliated Hospital of Zhejiang University School of Medicine (13)	Jianwei Zhou
Sir Run Run Shaw Hospital, Zhejiang University School of Medicine (14)	Guanghui Song
Taizhou First People's Hospital (15)	Zhu Danyang
Zhoushan Maternity and Infant Hospital (16)	Shi Xiao

© 2023 Che X et al. *JAMA Network Open.*

eAppendix 2. Inclusion Criteria and Exclusion Criteria

Inclusion criteria:

1. Women had a diagnosis of adenomyosis by B-ultrasound or pelvic MRI examination and the volume of their uterus were less than those with 10 weeks of pregnancy
2. Women with 10-cm visual analogue scale (VAS) of adenomyosis-associated pain > 0 point with or without menorrhagia (PBAC≥100 points)
3. Women between 18 and 50 years of age who currently have no childbearing requirement
4. Women who had normal cervical cytology test
5. Women who agreed to choose a barrier method of contraception
6. Women who agreed to adhere to the study requirements and signed the informed consent
7. Women who were not postmenopausal

Exclusion criteria:

1. Patients with HB < 90G/L
2. Patients with undiagnosed abnormal vaginal bleeding or endometrial lesions
3. Patients who were preparing to give birth when taking the medication or within 6 months of stopping the medication
4. Patients with malignant tumors (including the reproductive system and other systems);
5. Patients with severe heart, liver, kidney disease and adrenocortical insufficiency
6. Patients who had abnormal clinical significance in the follow-up laboratory test
7. Patients who had a known allergy to mifepristone, or related compounds
8. Patients with any other diseases or conditions that may cause the study drug to alter absorption, accumulate excessively, affect metabolism, or change the excretion pattern
9. Patients with clinically significant depression within the current or most recent year
10. Patients who regularly took analgesics due to other underlying diseases
11. Patients used the drugs, such as Ketoconazole, itraconazole, erythromycin, rifampicin, corticosteroids (hydrocortisone, prednisone, dexamethasone, etc.), and some anticonvulsants (phenytoin, phenobarsbital, carbamazepine, etc.), griseofulvin, non-steroidal anti-inflammatory drugs (aspirin, acetaminophen, etc.) and couldn't be stopped during the study
12. Patients currently used any other hormone drugs, including: a. use of GnRH agonists within 6 months before the screening period; b. use of progestins or danazol and other long-acting hormones within 3 months before the screening period; c. use of oral contraceptive-like short-acting hormones within one month before the screening period;
13. Patients who participated in other clinical trials within 3 months before the screening, or who were considered inappropriate to participate in the study by other investigators.

© 2023 Che X et al. *JAMA Network Open.*

eAppendix 3. Method of Sample Size Calculation

The sample-size calculation was based on the change of average score of VAS and we assumed that the VAS score decreased from 8.49 to 2.01 as previous studies showed [1]. However, there is not enough evidence about the effect of placebo for adenomyosis patients, since the efficacy results were different in several similar studies. According to the conservative principle in sample size calculation, the decrease of the VAS score in the placebo group has been assumed as 5.24 and 6.48 for the mifepristone group. The baselines of the VAS score of both groups have been assumed as 8.49 as well. To detect a significant difference between mifepristone therapy and placebo, 60 subjects per group were needed (α=0.05, β=0.1).

REFERENCES

1. L, L. Effect observation on different doses of mifepristone in the treatment of adenomyosis, China Prac Med, 2017; 32: 95-7.

© 2023 Che X et al. *JAMA Network Open.*

eTable 1. Change in VAS After Mifepristone Treatment

Mean change from baseline in VAS score after treatment (PPS [a], FAS [b])（Means ±SD）[c]						
Period of treatment	PPS		P value	FAS		P value
	Mifepristone	Placebo		Mifepristone	Placebo	
4 weeks	-6.37±2.25	-0.98±1.66	<.001	-6.37±2.25	-1.05±1.89	<.001
8 weeks	-6.65±1.84	-1.14±1.86	<.001	-6.65±1.84	-1.14±1.86	<.001
12 weeks	-6.63±1.92	-0.95±1.75	<.001	-6.63±1.92	-0.95±1.75	<.001
Mean change from baseline in VAS score after treatment (PPS-MI, FAS-MI)（Means ±SD）[d]						
Period of treatment	PPS-MI		P value	FAS-MI		P value
	Mifepristone	Placebo		Mifepristone	Placebo	
4 weeks	-6.37±2.25	-0.98±1.66	<.001	-6.38±2.24	-1.05±1.89	<.001
8 weeks	-6.48±2.05	-0.95±1.97	<.001	-6.49±2.04	-0.93±2.10	<.001
12 weeks	-6.44±2.08	-0.74±1.91	<.001	-6.45±2.07	-0.70±2.03	<.001

[a] PPS population is defined as all randomized patients who received at least one does of investigational product and have valid VAS assessments at both baseline and post-baseline.

[b] FAS population included the eligible subjects who were randomized.

[c] Primary analysis was performed with missing values of VAS score after treatment imputed as nonresponses.

[d] Primary analysis was repeated with missing values of VAS score after treatment imputed using the multiple imputation (MI) method.

© 2023 Che X et al. *JAMA Network Open.*

eTable 2. Total Effective Rate for Dysmenorrhea After Mifepristone Treatment

Total effective rate for dysmenorrhea after treatment (PPS[b], FAS[c]) n (%)[d]						
Period of treatment	PPS		P value	FAS		P value
	Mifepristone	Placebo		Mifepristone	Placebo	
4 weeks	56 (94.92%)	14 (22.22%)	<.001	56 (91.80%)	15 (23.08%)	<.001
8 weeks	57 (96.61%)	13 (20.63%)	<.001	57 (93.44%)	13 (20.00%)	<.001
12 weeks	56 (94.92%)	15 (23.81%)	<.001	56 (91.80%)	15 (23.08%)	<.001
**Total effective rate for dysmenorrhea after treatment (PPS-MI, FAS-MI) n (%)[e]**						
Period of treatment	PPS-MI		P value	FAS-MI		P value
	Mifepristone	Placebo		Mifepristone	Placebo	
4 weeks	56 (94.92%)	14 (22.22%)	<.001	58 (95.08%)	15 (23.08%)	<.001
8 weeks	58 (98.31%)	13 (20.63%)	<.001	60 (98.36%)	14 (21.54%)	<.001
12 weeks	58 (98.31%)	16 (25.40%)	<.001	60 (98.36%)	17 (26.15%)	<.001

[a] Definition of total effective rate for dysmenorrhea was the percentage of patients with reduction of ≥30% from baseline in VAS score after treatment.

[b] PPS population is defined as all randomized patients who received at least one does of investigational product and have valid VAS assessments at both baseline and post-baseline.

[c] FAS population included the eligible subjects who were randomized.

[d] Primary analysis was performed with missing values of VAS score after treatment imputed as nonresponses.

[e] Primary analysis was repeated with missing values of VAS score after treatment imputed using the multiple imputation (MI) method.

© 2023 Che X et al. *JAMA Network Open.*

eTable 3. Complete Remission Rate for Dysmenorrhea After Mifepristone Treatment

Complete remission rate for dysmenorrhea (PPS [b], FAS [c]) n (%) [d]						
Period of treatment	PPS		P value	FAS		P value
	Mifepristone	Placebo		Mifepristone	Placebo	
4 weeks	54 (91.53%)	5 (7.94%)	<.001	54 (88.52%)	6 (9.23%)	<.001
8 weeks	55 (93.22%)	6 (9.52%)	<.001	55 (90.16%)	6 (9.23%)	<.001
12 weeks	54 (91.53%)	4 (6.35%)	<.001	54 (88.52%)	4 (6.15%)	<.001
**Complete remission rate for dysmenorrhea (PPS-MI, FAS-MI) n (%) [e]**						
Period of treatment	PPS-MI		P value	FAS-MI		P value
	Mifepristone	Placebo		Mifepristone	Placebo	
4 weeks	54 (91.53%)	5 (7.94%)	<.001	54 (88.52%)	6 (9.23%)	<.001
8 weeks	55 (93.22%)	6 (9.52%)	<.001	55 (90.16%)	6 (9.23%)	<.001
12 weeks	54 (91.53%)	4 (6.35%)	<.001	54 (88.52%)	4 (6.15%)	<.001

[a] Definition of the complete remission rate for dysmenorrhea was the percentage of patients with 0 of VAS score after treatment.

[b] PPS population is defined as all randomized patients who received at least one does of investigational product and have valid VAS assessments at both baseline and post-baseline.

[c] FAS population included the eligible subjects who were randomized.

[d] Primary analysis was performed with missing values of VAS score after treatment imputed as nonresponses.

[e] Primary analysis was repeated with missing values of VAS score imputed using the multiple imputation (MI) method.

© 2023 Che X et al. *JAMA Network Open.*

eTable 4. Change in PBAC for Heavy Menstrual Bleeding After Mifepristone Treatment

Mean change from baseline in PBAC score after treatment (PPS [b], FAS [c]) (Means±SD) [d]						
Period of treatment	PPS		P value	FAS		P value
	Mifepristone	Placebo		Mifepristone	Placebo	
4 weeks	-258.08±168.32	-58.58±103.97	<.001	-258.08±168.32	-59.78±102.78	<.001
8 weeks	-258.08±168.32	-77.97±129.94	<.001	-258.08±168.32	-77.97±129.94	<.001
12 weeks	-257.89±169.60	-80.06±119.82	<.001	-257.89±169.60	-80.06±119.82	<.001
Mean change from baseline in PBAC score after treatment (PPS-MI, FAS-MI) (Means±SD) [e]						
Period of treatment	PPS-MI		P value	FAS-MI		P value
	Mifepristone	Placebo		Mifepristone	Placebo	
4 weeks	-258.08±168.32	-58.58±103.97	<.001	-256.33±167.21	-59.78±102.78	<.001
8 weeks	-258.08±168.32	-74.06±127.97	<.001	-255.96±167.28	-74.21±126.58	<.001
12 weeks	-257.43±167.48	-76.96±115.52	<.001	-255.90±166.25	-77.24±114.14	<.001

[a] The menstrual blood loss was analyzed with the pictorial blood loss assessment chart (PBAC). A PBAC score of 100 correlated with more than 80 ml of blood loss, which was defined as heavy menstrual bleeding.

[b] PPS population is defined as all randomized patients who received at least one does of investigational product and have valid PBAC assessments at both baseline and post-baseline.

[c] FAS population included the eligible subjects who were randomized.

[d] Primary analysis was performed with missing values of PBAC score after treatment imputed as nonresponses.

[e] Primary analysis was repeated with missing values of PBAC score after treatment imputed using the multiple imputation (MI) method.

© 2023 Che X et al. *JAMA Network Open.*

eTable 5. Total Effective Rate for Heavy Menstrual Bleeding After Mifepristone Treatment

Total effective rate for heavy menstrual bleeding after treatment (PPS [b], FAS [c]) n (%) [d]						
Period of treatment	PPS		P value	FAS		P value
	Mifepristone	Placebo		Mifepristone	Placebo	
4 weeks	39 (100.00%)	9 (25.00%)	<.001	39 (97.50%)	10 (27.03%)	<.001
8 weeks	39 (100.00%)	13 (36.11%)	<.001	39 (97.50%)	13 (35.14%)	<.001
12 weeks	38 (97.44%)	14 (38.89%)	<.001	38 (95.00%)	14 (37.84%)	<.001
Total effective rate for heavy menstrual bleeding after treatment (PPS-MI, FAS-MI) n (%) [e]						
Period of treatment	PPS-MI		P value	FAS-MI		P value
	Mifepristone	Placebo		Mifepristone	Placebo	
4 weeks	39 (100.00%)	9 (25.00%)	<.001	40 (100.0%)	10 (27.03%)	<.001
8 weeks	39 (100.00%)	13 (36.11%)	<.001	40 (100.0%)	14 (37.84%)	<.001
12 weeks	39 (100.00%)	15 (41.67%)	<.001	39 (97.50%)	16 (43.24%)	<.001

[a] Definition of total effective rate for heavy menstrual bleeding was the percentage of patients with reduction of ≥30% from baseline in PBAC score after treatment.

[b] PPS population is defined as all randomized patients who received at least one does of investigational product and have valid PBAC assessments at both baseline and post-baseline.

[c] FAS population included the eligible subjects who were randomized.

[d] Primary analysis was performed with missing values of PBAC score after treatment imputed as nonresponses.

[e] Primary analysis was repeated with missing values of PBAC score after treatment imputed using the multiple imputation (MI) method.

© 2023 Che X et al. *JAMA Network Open.*

eTable 6. Complete Remission Rate for Heavy Menstrual Bleeding After Treatment

Complete remission rate for heavy menstrual bleeding (PPS [b], FAS [c]) n (%) [d]						
Period of treatment	PPS		P value	FAS		P value
	Mifepristone	Placebo		Mifepristone	Placebo	
4 weeks	39 (100.00%)	2 (5.56%)	<.001	39 (97.50%)	3 (8.11%)	<.001
8 weeks	39 (100.00%)	2 (5.56%)	<.001	39 (97.50%)	2 (5.41%)	<.001
12 weeks	36 (92.31%)	2 (5.56%)	<.001	36 (90.00%)	2 (5.41%)	<.001
**Complete remission rate for heavy menstrual bleeding (PPS-MI, FAS-MI) n (%) [e]**						
Period of treatment	PPS-MI		P value	FAS-MI		P value
	Mifepristone	Placebo		Mifepristone	Placebo	
4 weeks	39 (100.00%)	2 (5.56%)	<.001	39 (97.50%)	3 (8.11%)	<.001
8 weeks	39 (100.00%)	2 (5.56%)	<.001	39 (97.50%)	2 (5.41%)	<.001
12 weeks	36 (92.31%)	2 (5.56%)	<.001	36 (90.00%)	2 (5.41%)	<.001

[a] Definition of complete remission rate for heavy menstrual bleeding was the percentage of patients with achieving amenorrhea after treatment.

[b] PPS population is defined as all randomized patients who received at least one does of investigational product and have valid PBAC assessments at both baseline and post-baseline.

[c] FAS population included the eligible subjects who were randomized.

[d] Primary analysis was performed with missing values of PBAC score after treatment imputed as nonresponses.

[e] Primary analysis was repeated with missing values of PBAC score after treatment imputed using the multiple imputation (MI) method.

© 2023 Che X et al. *JAMA Network Open.*

eTable 7. Changes From Baseline in Hb, CA125, PLT and Uterine Volume After Mifepristone Treatment

The changes in HB, CA125, PLT and uterine volume from baseline to 12 weeks treatment (PPS [b], FAS[c]) (Mean ±SD)						
End Point	PPS		P value	FAS		P value
	Mifepristone	Placebo		Mifepristone	Placebo	
HB (g/L) [d]	21.26±13.83	4.81±9.70	<.001	21.26±13.83	4.81±9.70	<.001
CA125 (U/mL)	-62.23±76.99	26.89±118.70	<.001	-62.23±76.99	26.89±118.70	<.001
PLT（10^9/L）	-28.87±54.30	2.06±41.78	0.001	-28.87±54.30	2.06±41.78	0.001
Uterine volume (cm^{3}) [e]	-29.32±39.34	18.39±66.46	<.001	-29.32±39.34	18.39±66.46	<.001

[a] Missing data were imputed as no response.

[b] PPS population is defined as all randomized patients who received at least one does of investigational product and have valid PBAC assessments at both baseline and post-baseline.

[c] FAS population included the eligible subjects who were randomized.

[d] The change from baseline in Hb is only for the patients with anemia. Definition of Anemia patients at baseline is the condition of having a lower-than 120 g/L quantity of hemoglobin.

[e] Uterine volume Uterine size was determined based on the diameters of three angles by transvaginal ultrasonography. The uterine volume was calculated with the following formula: 0.52×length× anteroposterior diameter× transverse diameter.

© 2023 Che X et al. *JAMA Network Open.*

eTable 8. Relevant Laboratory Evaluations Changed From Normal to Abnormal After Mifepristone Treatment

laboratory evaluations	mifepristone	placebo	P value
	N=59	N=64	
Alanine aminotransferase n [d]	57	62	0.0273[a]
Abnormal after treatment n (%)	7 (12.28%)	1 (1.61%)	
Normal after treatment n (%)	50 (87.72%)	61 (98.39%)	
Aspartate aminotransferase n	58	62	0.0516[b]
Abnormal after treatment n (%)	4 (6.90%)	0 (0.00%)	
Normal after treatment n (%)	54 (93.10%)	62(100.00%)	
Follicular estrogen hormone n	43	41	0.7048[c]
Abnormal after treatment n (%)	9 (20.93%)	10 (24.39%)	
Normal after treatment n (%)	34 (79.07%)	31 (75.61%)	
Luteinizing hormone n	56	52	0.0780[c]
Abnormal after treatment n (%)	26 (46.43%)	19 (30.65%)	
Normal after treatment n (%)	30 (53.57%)	43 (69.35%)	
Total cholesterol n	36	36	1.0000[b]
Abnormal after treatment n (%)	7 (19.44%)	6 (16.67%)	
Normal after treatment n (%)	29 (80.56%)	30 (83.33%)	

© 2023 Che X et al. *JAMA Network Open.*

laboratory evaluations	mifepristone N=59	placebo N=64	P value
Triglyceride n	34	37	0.4669[b]
Abnormal after treatment n (%)	5 (14.71%)	3 (8.11%)	
Normal after treatment n (%)	29 (85.29%)	34 (91.89%)	
Low-density lipoprotein n	37	44	0.7251[b]
Abnormal after treatment n (%)	5 (13.51%)	4 (9.09%)	
Normal after treatment n (%)	32 (86.49%)	40 (90.91%)	
Creatinine n	58	59	0.4957[b]
Abnormal after treatment n (%)	1 (1.72%)	0 (0.00%)	
Normal after treatment n (%)	57 (98.28%)	59(100.00%)	
Total bilirubin n	56	59	0.6797[b]
Abnormal after treatment n (%)	2 (3.57%)	4 (6.78%)	
Normal after treatment n (%)	54 (96.43%)	55 (93.22%)	

[a] Safety set (SS) population was defined as the subjects with at least one dose of the product intervention.

[b] P value obtained by Fisher's exact probability method.

[c] P-value obtained by Pearson's chi-square test.

[d] N refers to the number of subjects whose clinical judgment of the test index is 'normal' at baseline. The calculation of the percentage is based on the corresponding indicator n.

© 2023 Che X et al. *JAMA Network Open.*

eTable 9. Changes in Liver Function in the 2 Groups During Treatment

Case Number	drug Group	Visit time (week)	Liver function index	Evaluation before treatment	Value after treatment(U/L)	Evaluation after treatment
01-0017	A	12	ALT	normal	49	mildly abnormal
01-0051	A	12	ALT	normal	41	mildly abnormal
08-0015	A	4	ALT	normal	54	mildly abnormal
	A	4	AST	normal	55	mildly abnormal
	A	12	ALT	normal	39	normal
	A	12	AST	normal	33	normal
08-0034	A	12	ALT	normal	56	mildly abnormal
09-0002	A	4	ALT	normal	54.7	mildly abnormal
	A	4	AST	normal	41.6	mildly abnormal
	A	12	ALT	normal	16.2	normal
	A	12	AST	normal	19.1	normal
12-0005	A	4	ALT	normal	42	mildly abnormal
15-0006	B	4	ALT	normal	79	mildly abnormal
	B	12	ALT	normal	75	mildly abnormal
15-0007	A	12	ALT	normal	50	mildly abnormal
	A	12	AST	normal	46	mildly abnormal
08-0010	A	4	AST	normal	46	mildly abnormal
	A	12	AST	normal	29	normal

[a] Safety set (SS) population was defined as the subjects with at least one dose of the product intervention. A. Mifepristone group; B. Placebo group; ALT. Alanine aminotransferase; AST. Aspartate aminotransferase.

© 2023 Che X et al. *JAMA Network Open.*

eTable 10. Comparison of the Changes of Endometrial Thickness Between the 2 Groups After 4 Weeks of Treatment

	Mifepristone (n=59)	Placebo (n=64)	P value[b]
Effective frequency of endometrial thickness	53	58	
Chang of endometrial thickness from baseline (monolayer)(cm) [c]	-0.04 (-0.15 - 0.05)	0 (-0.135 - 0.05)	0.51
The decrease in endometrial thickness from baseline（%）[d]	-10 (-41.86 - 16.67)	0 (-33.33 - 33.33)	0.23

[a] Safety set (SS) population was defined as the subjects with at least one dose of the product intervention.

[b] P value obtained by rank sum test.

[c] Endometrial thickness (monolayer) was detected by transvaginal ultrasonography.

[d]The calculation formula of "endometrial thickness decrease from baseline (%)" is ((4th week-baseline) / baseline) *100%, this indicator represents the increase (decrease) of endometrial thickness after 4 weeks of treatment percentage, using Median (Q1, Q3) for statistical description.

© 2023 Che X et al. *JAMA Network Open.*

eTable 11. Comparison of the Change of Endometrial Thickness Between the 2 Groups After 12 Weeks of Treatment

	Mifepristone (n=59)	Placebo (n=64)	P value[b]
Effective frequency of endometrial thickness	58	62	
Chang of endometrial thickness from baseline (monolayer)(cm) [c]	-0.05 (-0.14, 0.05)	0.05 (-0.05, 0.15)	0.03
The decrease in endometrial thickness from baseline（%）[d]	-14.58 (-37.21, 26.66)	14.29 (-21.88, 44.44)	0.03

[a] Safety set (SS) population was defined as the subjects with at least one dose of the product intervention.

[b] P value obtained by rank sum test.

[c] Endometrial thickness (monolayer) was detected by transvaginal ultrasonography.

[d]The calculation formula of "endometrial thickness decrease from baseline (%)" is ((12th week-baseline) / baseline) *100%, this indicator represents the increase (decrease) of endometrial thickness after 12 weeks of treatment percentage, using Median (Q1, Q3) for statistical description.

© 2023 Che X et al. *JAMA Network Open.*

eTable 12. Comparison of the Endometrial Thickness Between the 2 Groups During the Treatment

Period of treatment	Mifepristone (N=59)	Placebo (N=64)	P value
0 week			
n(%)	58(98.31%)	62(96.88%)	
endometrial thickness (cm) [b]	0.36±0.13	0.34±0.15	0.24
4 weeks			
n(%)	53(89.83%)	58(90.63%)	
endometrial thickness (cm)	0.30±0.13	0.30±0.11	0.59
12 weeks			
n(%)	53(89.83%)	51(79.69%)	
endometrial thickness (cm)	0.33±0.19	0.38±0.19	0.11

[a] Safety set (SS) population was defined as the subjects with at least one dose of the product intervention.

[b] Endometrial thickness (monolayer) was detected by transvaginal ultrasonography.

© 2023 Che X et al. *JAMA Network Open.*

eFigure 1. Flowchart of Study Enrollment

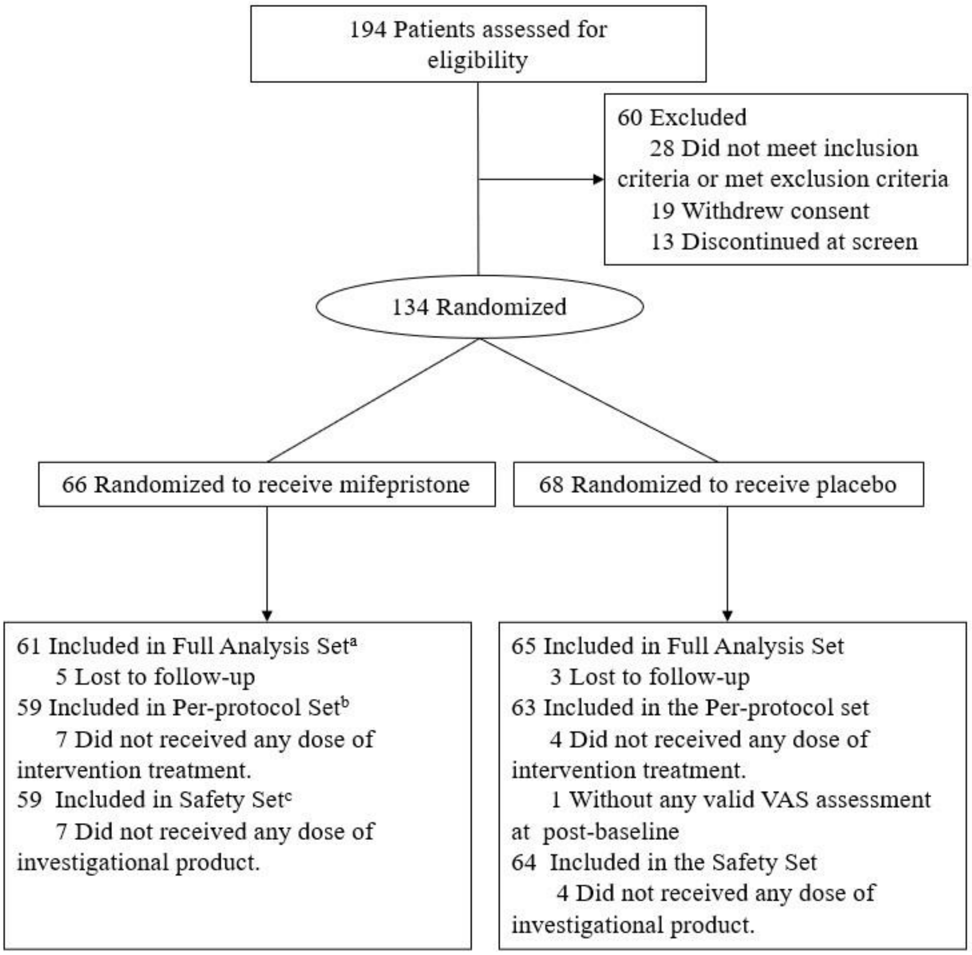

[a]The full analysis set is defined as all randomized patients. Patients without any valid data at baseline will be excluded. [b]The per-protocol set is defined as all randomized patients who received at least one dose of the investigational product and had valid VAS assessments at both baseline and post baseline. [c]The safety set is defined as all randomized patients who received at least one dose of the investigational product.

© 2023 Che X et al. *JAMA Network Open.*

eFigure 2. Levels of FSH, LH and E2 Between the 2 Groups at Different Periods of Treatment

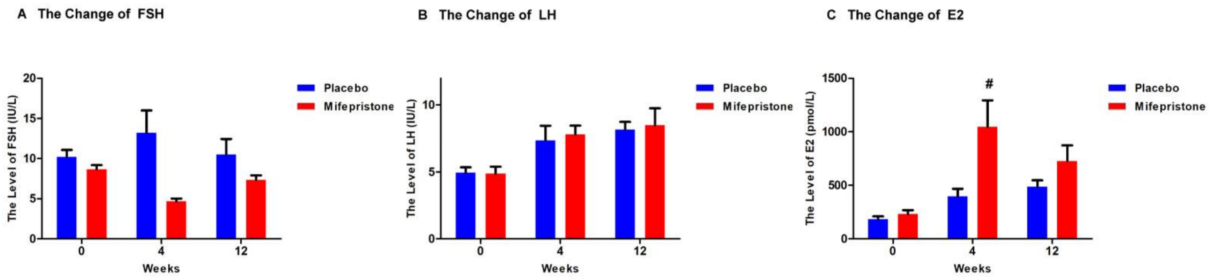

There are no significant differences of FSH and LH between mifepristone groups and placebo groups during the treatment. E2 was significantly increased in mifepristone group as compared with placebo group after 4-week treatment (P< 0.05) and there no significant differences after 12-week treatment. I bars indicate standard errors.

© 2023 Che X et al. *JAMA Network Open.*

eFigure 3. Changes in Endometrial Thickness and BMI Between the 2 Groups at Different Periods of Treatment

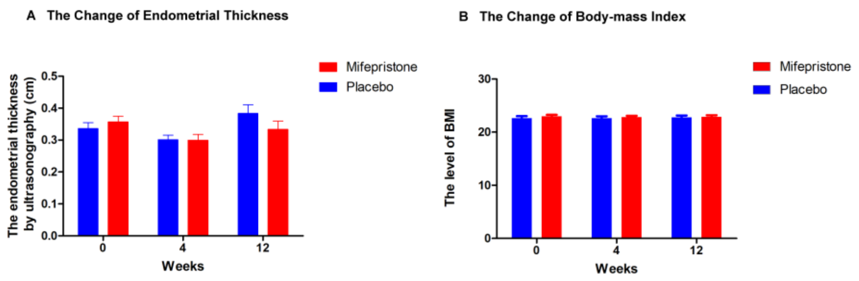

Panel A shows the levels of endometrial thickness after mifepristone treatment tended to be thin while there was no significant between the two groups. Panel B shows there was no significant differences for body-mass index between two groups.

© 2023 Che X et al. *JAMA Network Open.*
