# Supplementary material for: Effect of Mifepristone vs Placebo for Treatment of Adenomyosis With Pain Symptoms: A Randomized Clinical Trial
Source: JAMA Netw Open. 2023 Jun 12;6(6):e2317860. doi: 10.1001/jamanetworkopen.2023.17860 (PMC10261993; doi:10.1001/jamanetworkopen.2023.17860)
Supplement: Supplement 3. — Data Sharing Statement [file jamanetwopen-e2317860-s003.pdf]

# Data Sharing Statement

Che. Effect of Mifepristone vs Placebo for Treatment of Adenomyosis With Pain Symptoms. *JAMA Netw Open*. Published June 12, 2023. doi:10.1001/jamanetworkopen.2023.17860

**Data**
**Data available:** No
